# Invasive breast cancer and breast cancer death after non-screen detected ductal carcinoma in situ from 1990 to 2018 in England: population based cohort study

**DOI:** 10.1136/bmj-2023-075498

**Published:** 2024-01-24

**Authors:** Gurdeep S Mannu, Zhe Wang, David Dodwell, John Broggio, Jackie Charman, Sarah C Darby

**Affiliations:** 1 Nuffield Department of Population Health, University of Oxford, Richard Doll Building, Old Road Campus, Oxford, UK; 2 Nuffield Department of Surgical Sciences, University of Oxford, Richard Doll Building, Old Road Campus, Oxford, UK; 3 National Disease Registration Service, NHS England, Birmingham, UK

## Abstract

**Objectives:**

To evaluate the long term risks of invasive breast cancer and death related to breast cancer after non-screen detected ductal carcinoma in situ. Risks for women in the general population and for women diagnosed with ductal carcinoma in situ via the screening programme were compared.

**Design:**

Population based cohort study.

**Setting:**

Data from the National Disease Registration Service.

**Participants:**

All 27 543 women in England who were diagnosed with ductal carcinoma in situ, outside the NHS breast screening programme, during 1990 to 2018.

**Main outcome measures:**

Incident invasive breast cancer and death caused by breast cancer.

**Results:**

By 31 December 2018, 3651 women with non-screen detected ductal carcinoma in situ had developed invasive breast cancer, more than four times higher than expected from national cancer incidence rates (ratio of observed to expected rate was 4.21 (95% conference interval 4.07 to 4.35)). The ratio of observed to expected rate of developing invasive breast cancer remained increased throughout follow-up among women aged <45-70 years. The 25 year cumulative risks of invasive breast cancer by age at diagnosis of ductal carcinoma in situ were 27.3% for <45 years, 25.2% for 45-49 years, 21.7% for 50-59 years, and 20.8% for 60-70 years. 908 women died of breast cancer, almost four times higher than that expected from breast cancer death rates in the general population (ratio of observed to expected rate 3.83 (3.59 to 4.09)). The ratio of observed to expected rate of mortality attributed to breast cancer remained increased throughout follow-up. The 25 year cumulative risks of breast cancer death by age at ductal carcinoma in situ diagnosis were 7.6% for <45 years, 5.8% for 45-49 years, 5.9% for 50-59 years, and 6.2% for 60-70 years. Among women aged 50-64 years, and therefore eligible for breast screening by the NHS, the ratio of observed to expected rate of invasive breast cancer in women with non-screen detected compared with screen detected ductal carcinoma in situ was 1.26 (95% conference interval 1.17 to 1.35), while the ratio for mortality from breast cancer was 1.37 (1.17 to 1.60). Among 22 753 women with unilateral ductal carcinoma in situ undergoing surgery, those who had mastectomy rather than breast conserving surgery had a lower 25 year cumulative rate of ipsilateral invasive breast cancer (mastectomy 8.2% (95% conference interval 7.0% to 9.4%), breast conserving surgery with radiotherapy 19.8% (16.2% to 23.4%), and breast conserving surgery with no radiotherapy recorded 20.6% (18.7% to 22.4%)). However, reductions did not translate into a lower 25 year cumulative rate of deaths attributable to breast cancer (mastectomy 6.5% (4.9% to 10.9%), breast conserving surgery with radiotherapy 8.6% (5.9% to 15.5%), breast conserving surgery with no radiotherapy recorded 7.8% (6.3% to 11.5%)).

**Conclusions:**

For at least 25 years after their diagnosis, women with non-screen detected ductal carcinoma in situ had higher long term risks of invasive breast cancer and breast cancer death than women in the general population. Additionally, they had higher long term risks than women with screen detected ductal carcinoma in situ. Mastectomy was associated with lower risks of invasive breast cancer than breast conserving surgery, even when accompanied by radiotherapy. However, risks of breast cancer death appeared similar for mastectomy, breast conserving surgery with radiotherapy, and breast conserving surgery with no radiotherapy recorded.

## Introduction

The increased incidence of ductal carcinoma in situ over recent decades has been attributed largely to the National Health Service (NHS) breast screening programme. Many women are, however, diagnosed with ductal carcinoma in situ outside the screening programme, either because they are not in the age range included in the screening programme, or because they did not respond to a screening invitation, or because their carcinoma arose during an interval between three-yearly screens. The risks of subsequent invasive breast cancer and breast cancer death among women with a diagnosis of non-screen detected ductal carcinoma in situ are unclear, as is the way in which the risks evolve over time. Hence, the optimal period of post-treatment follow-up and frequency of surveillance imaging following a diagnosis of non-screen detected ductal carcinoma in situ are uncertain.

Many diagnoses of ductal carcinoma in situ occur as a result of screening, which has led to concerns that ductal carcinoma in situ may be overtreated in some women, something that is being investigated in various ongoing randomised trials evaluating non-operative management of ductal carcinoma in situ.^1^ ^2^ ^3^ However, alongside these concerns, it is also the case that the rates of invasive breast cancer and breast cancer death in women with screen detected ductal carcinoma in situ are more than double those of women in the general population and remain raised for at least 20 years after diagnosis.^4^ A greater understanding of the risks following non-screen detected ductal carcinoma in situ would provide insights into the natural history of ductal carcinoma in situ and into whether concerns regarding overtreatment of screen detected ductal carcinoma in situ are warranted.

To provide further information on the long term consequences of ductal carcinoma in situ diagnosed outside the screening programme, we undertook a population based, observational study characterising the risks of invasive breast cancer and breast cancer death among all women diagnosed with non-screen detected ductal carcinoma in situ in England. We compared the risks for these women with those for the general population and for women diagnosed with screen detected ductal carcinoma in situ. We also investigated patient, tumour, and treatment related factors that are associated with these endpoints in women with non-screen detected ductal carcinoma in situ.

## Methods

### Study population and data

The NHS breast screening programme,^5^ began in 1988 and achieved national coverage in 1993. The service is centralised in which women (specifically, people registered with a general practitioner as female) in specified age groups are sent personal letters every three years inviting them to attend an appointment for screening mammography in one of 78 breast screening units across England. Initially, women aged 50-64 years were invited. However, from 2003 the age range was extended to include women aged 65-70 years. The attendance rate for women invited for screening has consistently been over 70%.^6^

Since the introduction of the NHS breast screening programme in England, all women diagnosed with either screen detected or non-screen detected ductal carcinoma in situ have been registered prospectively as such. These registrations were initially with the Regional Cancer Registries, but these have now been unified into the National Disease Registration Service.^7^ Registrations are routinely linked at the patient level with other information on the same woman, including registrations of other cancers, the date of any emigration, and the date and cause of death, if relevant. For this study, the National Disease Registration Service compiled a dataset that included information on 82 009 women diagnosed between 1990 and 2018 and followed up to December 2018. This was the most recent dataset available for cancer registrations at the time of applying. The dataset was then de-personalised and released to the investigators in the Nuffield Department of Population Health at the University of Oxford for analysis. 

The dataset received by these investigators included, for each woman, information on patient related factors (date of ductal carcinoma in situ diagnosis, age at diagnosis, laterality of ductal carcinoma in situ, whether or not the disease was screen detected, information about the treatment given (surgery and radiotherapy) where recorded, and any prior cancer diagnoses), date and site of any subsequent breast cancer registrations, date of emigration, and date and cause of death, if relevant. To provide reassurance that women included in the study had, in fact, been diagnosed initially with ductal carcinoma in situ rather than invasive breast cancer, the textual pathology reports stored by the National Disease Registration Service for a random sample of 130 women for whom an invasive breast cancer or breast cancer death was subsequently reported were examined by two clinicians (GSM and DD); no cases were found in which the initial diagnosis had been invasive breast cancer rather than ductal carcinoma in situ. The study was approved by Yorkshire and The Humber-Leeds East research ethics committee (16/YH/0209), the NHS breast screening programme research committee, and Public Health England’s office for data release (ODR1516_225).

### Data analysis

Data for women with ductal carcinoma in situ were separated into those with screen detected diagnoses and those with non-screen detected diagnoses. We excluded any woman recorded with a previous invasive cancer (other than non-melanoma skin cancer), aged ≥90 years at diagnosis, or with no histological confirmation of ductal carcinoma in situ. Women with either a record of invasive breast cancer or a record of having received chemotherapy within six months of ductal carcinoma in situ diagnosis were also excluded. Further exclusions included women with inconsistent records and those with less than six months of follow-up. The analysis focused on women with non-screen detected ductal carcinoma in situ, apart from a comparison of the risks of invasive breast cancer and breast cancer death between women with screen detected and those with non-screen detected ductal carcinoma in situ diagnosed during 1990-2018 at ages 50-64 years. All women in this age group were eligible for screening within the NHS breast screening programme throughout this calendar period. Further results for women with screen detected ductal carcinoma in situ have been given elsewhere.^4^

We considered women from six months after their diagnosis of ductal carcinoma in situ (so that the most recent diagnosis date considered was 30 June 2018) until the earliest of diagnosis of invasive breast cancer or death, loss to follow-up, the woman’s 90th birthday or 31 December 2018 (see supplementary text S1, sections A-C). We calculated observed numbers and observed rates of invasive breast cancer, breast cancer death, and non-breast cancer death. We also calculated cumulative expected numbers and expected risks for these endpoints, using cancer incidence rates for England and mortality rates for England and Wales both in five-year age groups and single calendar years. Confidence intervals for rates in the study population and for the ratios of observed to expected events and event rates assumed that the numbers of observed deaths had a Poisson distribution and that the numbers of expected deaths were fixed. Variability in the ratio of observed to expected death rates was studied using Poisson regression (supplementary text S1, section D). Tests for trend or heterogeneity in the ratio across individual characteristics were conducted using a likelihood ratio test, as were tests for an interaction between two characteristics. The ratios of observed rates in different groups of women were studied in a similar fashion using Poisson regression (supplementary text S1, section H), and cumulative rates assumed that the numbers of observed events in all the groups of women studied followed a Poisson distribution (supplementary text S1, section I). We calculated cumulative observed risks of invasive breast cancer and of breast cancer death based on the annual event rates and we took competing causes of death into account using five-year age specific mortality rates for England and Wales (supplementary text S1, sections E-G). Calculations were performed using Stata statistical software version 15.1 (StataCorp, College Station, TX, USA) and R (version 4.2.2).

### Patient and public involvement

This study comprises a statistical analysis conducted on routinely collected patient data that had been depersonalised. Two patients representing the organisation Independent Cancer Patients’ Voice were involved as research partners. They advised on which analyses would be informative to patients. They also reviewed and commented on the main findings in the manuscript via face-to-face meetings, teleconferences, and email. They have agreed to help with dissemination of the findings.

## Results

### Characteristics of women with non-screen detected ductal carcinoma in situ

**Fig 1:**
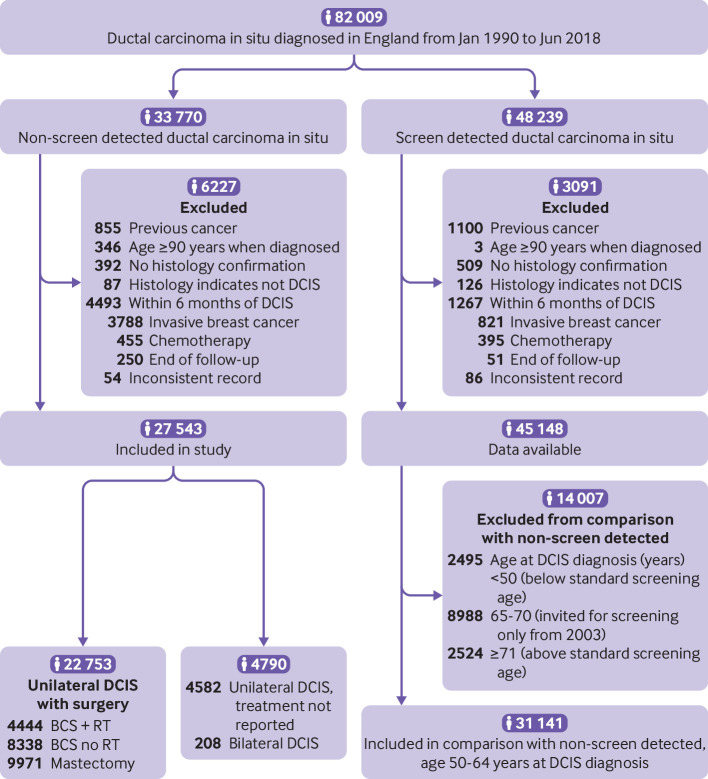
Derivation of study population. DCIS=ductal carcinoma in situ; BCS+RT=breast conserving surgery, radiotherapy recorded; BCS no RT=breast conserving surgery, radiotherapy not recorded

By 30 June 2018, a total of 27 543 women in England had been diagnosed with non-screen detected ductal carcinoma in situ as their first cancer and were included in the study (fig 1). Of these, 22 753 women had unilateral ductal carcinoma in situ and were treated with surgery (4444 (19.5%) with breast conserving surgery with radiotherapy, 8338 (36.7%) with breast conserving surgery without a record of radiotherapy, 9971 (43.8%) mastectomy). Among the remaining 4790 women, 4582 (95.7%) had unilateral ductal carcinoma in situ, while 208 (4.3%) were diagnosed with bilateral ductal carcinoma in situ. The number of women diagnosed per year increased with each calendar year (P_heterogeneity_<0.001) (table 1). Over half the women were diagnosed when aged younger than 50 years or at least 71 years, and therefore would not have received screening invitations (table 1).

**Table 1:** Characteristics of 27 543 women in England diagnosed with non-screen detected ductal carcinoma in situ (DCIS) between 1990 and 2018 and their status in December 2018. The table shows number of women in each category and the column percentage

Characteristics	Bilateral DCIS	Unilateral DCIS						
		BCS + RT	BCS no RT	Mastectomy	Unknown*	P value†	Total	
Calendar year of DCIS diagnosis:								
1990-99	52 (25.0)	749 (16.9)	2534 (30.4)	2060 (20.7)	2598 (56.7)	<0.001	7993 (29.0)	
2000-09	47 (22.6)	1461 (32.9)	2889 (34.6)	4013 (40.2)	904 (19.7)	—	9314 (33.8)	
2010-18	109 (52.4)	2234 (50.3)	2915 (35.0)	3898 (39.1)	1080 (23.6)	—	10 236 (37.2)	
Age at DCIS diagnosis, years:								
<45	34 (16.3)	867 (19.5)	1280 (15.4)	2515 (25.2)	577 (12.6)	<0.001	5273 (19.1)	
45-49	43 (20.7)	899 (20.2)	1353 (16.2)	1821 (18.3)	514 (11.2)	—	4630 (16.8)	
50-59	55 (26.4)	1166 (26.2)	2092 (25.1)	2178 (21.8)	1417 (30.9)	—	6908 (25.1)	
60-70	36 (17.3)	910 (20.5)	1770 (21.2)	1698 (17.0)	1019 (22.2)	—	5433 (19.7)	
≥71	40 (19.2)	602 (13.5)	1843 (22.1)	1759 (17.6)	1055 (23.0)	—	5299 (19.2)	
Laterality of DCIS:								
Left	—	2282 (51.4)	4240 (50.9)	5184 (52.0)	2175 (47.5)	0.81	13 881 (50.4)	
Right	—	2125 (47.8)	3878 (46.5)	4669 (46.8)	2001 (43.7)	—	12 673 (46.0)	
Bilateral‡	208 (100.0)	—	—	—	—	—	208 (0.8)	
Unknown‡	—	37 (0.8)	220 (2.6)	118 (1.2)	406 (8.9)	—	781 (2.8)	
Length of follow-up since DCIS diagnosis, years:								
<5	72 (34.6)	1520 (34.2)	2055 (24.6)	2561 (25.7)	1143 (24.9)	—	7351 (26.7)	
5-9	55 (26.4)	1112 (25.0)	1978 (23.7)	2581 (25.9)	704 (15.4)	—	6430 (23.3)	
10-19	52 (25.0)	1370 (30.8)	2887 (34.6)	3708 (37.2)	1044 (22.8)	—	9061 (32.9)	
≥20	29 (13.9)	442 (9.9)	1418 (17.0)	1121 (11.2)	1691 (36.9)	—	4701 (17.1)	
Invasive breast cancer diagnosed by 31 December 2018:								
Ipsilateral	—	288 (6.5)	825 (9.9)	435 (4.4)	402 (8.8)	—	1950 (7.1)	
Contralateral	—	157 (3.5)	351 (4.2)	413 (4.1)	182 (4.0)	—	1103 (4.0)	
Unknown	21 (10.1)	60 (1.4)	125 (1.5)	133 (1.3)	259 (5.7)	—	598 (2.2)	
Vital status on 31 December 2018:								
Alive	173 (83.2)	3899 (87.7)	6695 (80.3)	8368 (83.9)	2992 (65.3)	—	22 127 (80.3)	
Emigrated§	3 (1.4)	69 (1.6)	143 (1.7)	154 (1.5)	109 (2.4)	—	478 (1.7)	
Dead	32 (15.4)	476 (10.7)	1500 (18.0)	1449 (14.5)	1481 (32.3)	—	4938 (17.9)	
Cause of death:								
Breast cancer	3 (1.4)	112 (2.5)	232 (2.8)	250 (2.5)	311 (6.8)	—	908 (3.3)	
Other causes	27 (13.0)	349 (7.9)	1250 (15.0)	1185 (11.9)	1139 (24.9)	—	3950 (14.3)	
Unknown cause	2 (1.0)	15 (0.3)	18 (0.2)	14 (0.1)	31 (0.7)	—	80 (0.3)	
Total	208 (100.0)	4444 (100.0)	8338 (100.0)	9971 (100.0)	4582 (100.0)	—	27 543 (100.0)	

BCS+RT=breast conserving surgery, radiotherapy recorded; BCS no RT=breast conserving surgery, radiotherapy not recorded; DCIS=ductal carcinoma in situ.

* Includes both women who did not have surgery and those who had surgery but for whom it was not recorded. Numbers of women with unknown treatment by year of diagnosis and age at diagnosis are in table S1.

† P values for heterogeneity tests.

‡ Bilateral and unknown excluded from heterogeneity tests.

§ See table S2 for numbers emigrated by age at diagnosis and calendar year of diagnosis.

By 31 December 2018, 9061 women had been followed up for 10-19 years and 4701 for more than 20 years, while 478 (1.7%) women had emigrated. A total of 908 women had died from breast cancer. Additionally, 4030 women had died from other or unknown causes, which is considerably fewer than expected from mortality rates in the general population (ratio of observed to expected death rate 0.73 (95% confidence interval 0.71 to 0.76), tables S3-4).

### Incidence of invasive breast cancer in women with non-screen detected ductal carcinoma in situ

By 31 December 2018, 3651 women with non-screen detected ductal carcinoma in situ had developed invasive breast cancer (1950 ipsilateral, 1103 contralateral, 598 unknown laterality) (table 1). The invasive breast cancer rate was 13.28 (95% confidence interval 12.85 to 13.71) per 1000 woman years overall. It varied with age at diagnosis of ductal carcinoma in situ (P_heterogeneity_<0.001) and was 14.80 per 1000 woman years at ages <45 years, 12.32 for 45-49 years, 11.37 for 50-59 years, 12.65 for 60-69 years, and 18.61 for ≥70 years (table S5).

The invasive breast cancer rate was more than four times that expected based on breast cancer incidence rates in the general population (observed to expected rate ratio 4.21 (95% confidence interval 4.07 to 4.35), P<0.001). The rate ratio also varied with age at ductal carcinoma in situ diagnosis (P_heterogeneity_<0.001) (table S5). The ratios for women by diagnosis age were 7.33 at <45 years, 4.28 at 45-49 years, 3.37 at 50-59 years, 3.49 at 60-70 years, and 4.58 at ≥71 years. The observed to expected rate ratios decreased steadily with time since ductal carcinoma in situ diagnosis (P_heterogeneity_<0.001) with values of 5.56 (95% confidence interval 5.29 to 5.84) at 0.5-4 years after diagnosis, 4.32 (4.07 to 4.59) after 5-9 years, 3.06 (2.86 to 3.27) after 10-19 years, and 2.50 (2.13 to 2.95) after ≥20 years.

**Fig 2:**
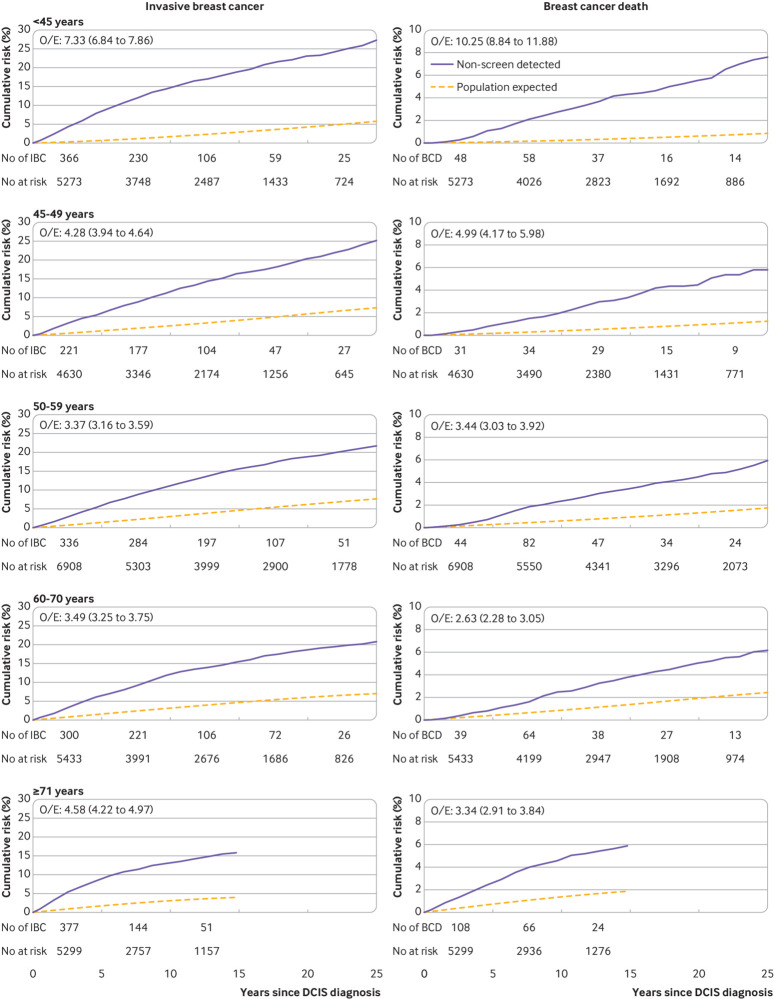
Cumulative risk of invasive breast cancer (left) and of death from breast cancer (right) in 27 543 women with non-screen detected DCIS by age at diagnosis of DCIS. O/E is ratio of observed and expected rates with 95% confidence interval. Cumulative risks take into account competing risks from other causes of death. Expected values are based on cancer incidence rates for England and mortality rates for England and Wales. For women aged ≥71 years at diagnosis cumulative risks have been calculated only up to 15 years since diagnosis as the study followed women only up to age 90 years. Cumulative risks and confidence intervals are in tables S9 & S10 (see also tables S5-S8). IBC=invasive breast cancer; BCD=breast cancer death; DCIS=ductal carcinoma in situ

The observed and expected cumulative risks of invasive breast cancer diverged with increasing time since diagnosis in every age at diagnosis group. The risks continued to diverge throughout follow-up and, by 25 years after ductal carcinoma in situ diagnosis, observed and expected cumulative risks of invasive breast cancer were 27.3% and 5.8% for <45 years, 25.2% and 7.3% for 45-49 years, 21.7% and 7.7% for 50-59 years, and 20.8% and 7.0% for 60-70 years (fig 2, left).

**Fig 3:**
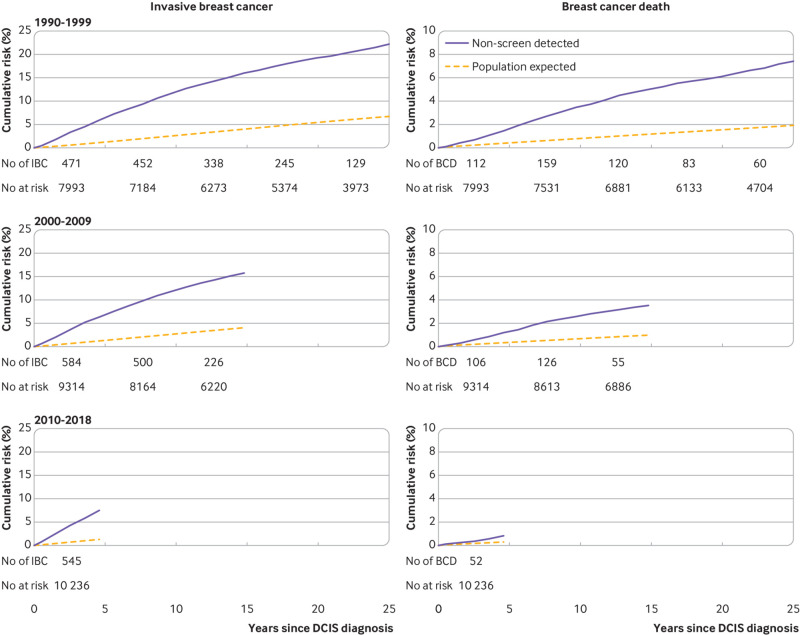
Cumulative risk of invasive breast cancer (left) and of death from breast cancer (right) in 27 543 women with non-screen detected DCIS by year of diagnosis of DCIS. Cumulative risks take into account competing risks from other causes of death. Expected values are based on cancer incidence rates for England and mortality rates for England and Wales. Cumulative risks and confidence intervals are in tables S11 & S12 (see also tables S5-S8). IBC=invasive breast cancer; BCD=breast cancer death, DCIS=ductal carcinoma in situ

No significant trend was found in the observed to expected rate ratio of invasive breast cancer with calendar year of ductal carcinoma in situ diagnosis after accounting for age at ductal carcinoma in situ diagnosis and time since diagnosis (P_trend_=0.45) (table S6). The cumulative observed and expected risks of invasive breast cancer diverged with increasing time since diagnosis of ductal carcinoma in situ in every calendar period of diagnosis (fig 3, left). By 25 years after ductal carcinoma in situ diagnosis, the cumulative observed risk of invasive breast cancer for women diagnosed before the year 2000 was 22.2% compared with 6.7% expected.

### Mortality from breast cancer in women with non-screen detected ductal carcinoma in situ

A total of 908 women with non-screen detected ductal carcinoma in situ died with breast cancer as the certified cause of death (table 1). The breast cancer death rate was 3.05 per 1000 woman years overall and, as for invasive breast cancer incidence, it varied with age at diagnosis of ductal carcinoma in situ (P_heterogeneity_<0.001) (table S7). The breast cancer death rate per 1000 woman years by diagnosis age was 2.98 for <45 years, 2.33 for 45-59 years, 2.49 for 50-59 years, 2.96 for 60-70 years, and 6.11 for ≥71 years.

Considering all women with non-screen-detected ductal carcinoma in situ, the observed breast cancer death rate was almost four times higher than that expected from mortality rates in the general population (observed to expected rate ratio 3.83 (95% confidence interval 3.59 to 4.09), (P_heterogeneity_<0.001)). This rate ratio varied with age at diagnosis of ductal carcinoma in situ: 10.25 for women diagnosed at age <45 years, 4.99 for 45-49 years, 3.44 for 50-59 years, 2.63 for 60-70 years, and 3.34 for ≥71 years (table S7). The ratio of the observed to the expected breast cancer death rate also varied with time since ductal carcinoma in situ diagnosis (P_heterogeneity_<0.001) with values of 3.59 (95% confidence interval 3.19 to 4.05) at 0.5-4 years after diagnosis, 4.72 (4.22 to 5.28) after 5-9 years, and 3.44 (3.05 to 3.88) after 10-19 years. The death rate remained markedly raised even after 20 years or more from diagnosis, (ratio of observed to expected death rates 3.34 (2.63 to 4.25).

The observed and expected cumulative risks of breast cancer death diverged with increasing time since diagnosis in every age at diagnosis group (fig 2, right). By 25 years after ductal carcinoma in situ diagnosis, the observed and expected cumulative risks were 7.6% and 0.9% for age <45 years at diagnosis, 5.8% and 1.2% for 45-49 years, 5.9% and 1.7% for 50‑59 years, and 6.2% and 2.4% for 60-70 years.

The observed cumulative risk of breast cancer death in women with non-screen detected ductal carcinoma in situ exceeded that expected in women in the general population in all three calendar periods studied (table S8). The ratio of the observed to the expected breast cancer death rate decreased with calendar year of diagnosis of ductal carcinoma in situ when time since diagnosis was considered (P_trend_<0.001). The ratio of the observed to the expected death rate during the period 0.5-4 years after diagnosis of ductal carcinoma in situ was 4.00 (95% confidence interval 3.32 to 4.81) for 1990-99 diagnoses, 3.81 (3.15 to 4.60) for 2000-09 diagnoses, and 2.69 (2.05 to 3.53), for 2010-18 diagnoses. In all three calendar periods, the observed and expected cumulative risks diverged with increasing time since diagnosis of ductal carcinoma in situ (fig 3, right). Among women diagnosed before 2000, the 25 year cumulative risk of breast cancer death was 7.4% (95% confidence interval 6.8% to 8.0%) compared with 1.9% expected in the general population; among women diagnosed during 2000-10, the 15 year cumulative risk was 3.5% (3.1% to 3.9%) compared with 1.0% expected; and among women diagnosed during 2010-18, the five year cumulative risk was 0.8% (0.6% to 1.0%) compared with 0.3% in the general population.

### Comparison between women with non-screen detected and screen detected ductal carcinoma in situ

A total of 9679 women were diagnosed with non-screen detected ductal carcinoma in situ at ages 50-64 years, that is, within the age range that was eligible for screening within the NHS breast screening programme throughout the study period. In the same age range, 31 141 women were diagnosed with screen detected ductal carcinoma in situ during the study period (fig 1). The proportion of women whose ductal carcinoma in situ was detected via screening increased with each calendar period (P_heterogeneity_<0.001) (table S13). Women with non-screen detected ductal carcinoma in situ tended to be younger when diagnosed than those with screen detected ductal carcinoma in situ: 40.4% versus 38.2% were aged 50-54 years, 31.0% versus 30.4% were aged 55-59 years, and 28.6% versus 31.4% were aged 60-64 years (P_heterogeneity_<0.001). Compared with the general population, fewer women lived in areas classified as “most deprived” in the index of multiple deprivation in both non-screen detected and screen detected groups, and more women lived in areas classified as “least deprived”. However, the distribution across the five index of multiple deprivation quintiles did not differ significantly between the two groups (P=0.43, table S13).

**Fig 4:**
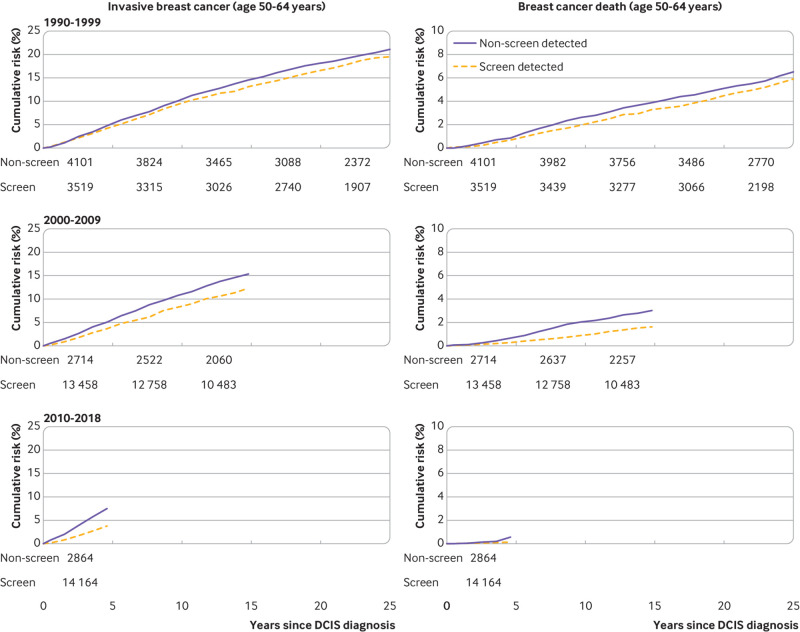
Cumulative risks of invasive breast cancer (left) and of death from breast cancer (right) in 9679 women with non-screen detected DCIS and 31 141 women with screen detected DCIS aged 50-64 years by year of diagnosis. The numbers of women at risk in screen detected and non-screen detected are given under each panel. Cumulative risks take into account competing risks from other causes of death. Cumulative risks and confidence intervals are in tables S16 and S17 (see also tables S13-S15). DCIS=ductal carcinoma in situ

The ratio of the invasive breast cancer rate among women with non-screen detected ductal carcinoma in situ to that of women with screen detected ductal carcinoma in situ was 1.26 (95% confidence interval 1.17 to 1.35) overall. The ratio tended to decrease with time since diagnosis of ductal carcinoma in situ within categories of calendar period (P<0.001) and increase with each calendar period category within time since diagnosis categories (P<0.001) (table S14). For women diagnosed during 2000-09, the cumulative invasive breast cancer risk at five years was 5.1% and at 15 years was 15.4% for non-screen detected ductal carcinoma in situ compared with 3.7% and 12.3% for screen detected ductal carcinoma in situ. For women diagnosed during 2010-18, the five year risks of invasive breast cancer were 7.5% for non-screen detected ductal carcinoma in situ compared with 3.8% for screen detected ductal carcinoma in situ (fig 4, left).

The overall ratio of the breast cancer death rate among women with non-screen detected compared with screen detected ductal carcinoma in situ after taking calendar period and time since diagnosis into account was 1.37 (95% confidence interval 1.17 to 1.60). This ratio tended to decrease with time since diagnosis after each calendar period of diagnosis was taken into account (P_trend_<0.001) but increased with calendar period after time since diagnosis was taken into account (P_trend_<0.001) (table S15). For women diagnosed during 2000-09, the cumulative risk of breast cancer death at five years after diagnosis was 0.7% and at 15 years it was 3.0% for women with non-screen detected ductal carcinoma in situ compared with 0.3% and 1.6% for women with screen detected ductal carcinoma in situ. For women diagnosed during 2010-18, the five year risk of breast cancer death was 0.6% for women with non-screen detected ductal carcinoma in situ compared with 0.1% for women with screen detected ductal carcinoma in situ (fig 4, right). Further results for women with screen detected ductal carcinoma in situ have been given elsewhere.^4^

### Treatments and outcomes in non-screen detected ductal carcinoma in situ

**Fig 5:**
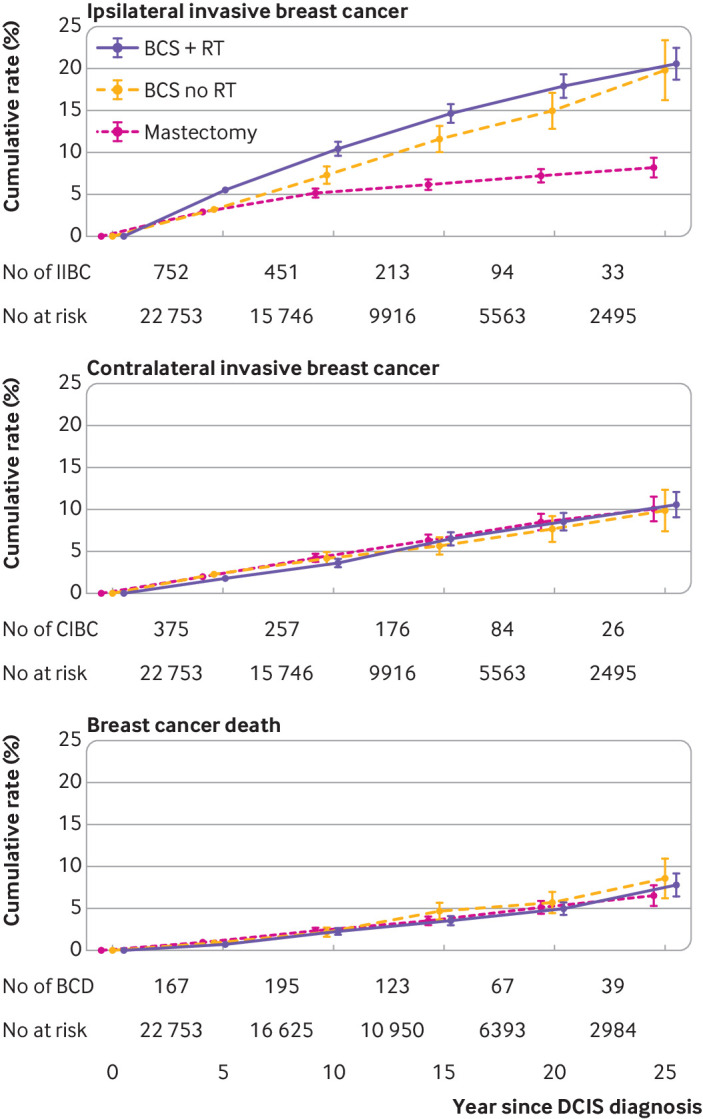
Cumulative incidence rates of ipsilateral and contralateral invasive breast cancer and cumulative rate of breast cancer death by recorded treatment and 95% confidence intervals in 22 753 women with non-screen detected unilateral DCIS during 1990-2018. IIBC=ipsilateral invasive breast cancer; CIBC=contralateral invasive breast cancer; BCD=breast cancer death; BCS+RT=breast conserving surgery, radiotherapy recorded; BCS no RT=breast conserving surgery, radiotherapy not recorded. Cumulative risks, confidence intervals, and significance tests are in table S1

Of the 22 753 women diagnosed with unilateral non-screen detected ductal carcinoma in situ and recorded as receiving surgery, invasive breast cancer rates differed according to whether a woman had breast conserving surgery with radiotherapy, breast conserving surgery without a record of radiotherapy, or mastectomy (P_heterogeneity_<0.001, table S18). Women who received breast conserving surgery but had no record of radiotherapy had higher cumulative incidence rates of ipsilateral invasive breast cancer than did women who received breast conserving surgery and radiotherapy during the first 10 years after diagnosis (fig 5, upper panel). After this point, the cumulative incidence rates in women treated with breast conserving surgery, with and without radiotherapy, converged, and by 25 years after diagnosis the cumulative invasive breast cancer rate was 20.6% (95% confidence interval 18.7% to 22.4%) after breast conserving surgery without a record of radiotherapy and 19.8% (16.2% to 23.4%) after conserving surgery with radiotherapy. Women receiving a mastectomy had lower rates of ipsilateral invasive breast cancer than women who received breast conserving surgery throughout follow-up and their cumulative invasive breast cancer rate at 25 years was 8.2% (7.0% to 9.4%). In contrast to ipsilateral invasive breast cancer, no significant heterogeneity between the three treatment modalities was found for contralateral invasive breast cancer (P_heterogeneity_=0.94) or breast cancer death (P_heterogeneity_=0.57). The 25 year cumulative breast cancer mortality rate was 8.6% (5.9% to 15.5%) for breast conserving surgery with radiotherapy, 7.8% (6.3% to 11.5%) for breast conserving surgery without radiotherapy, and 6.5% (4.9% to 10.9%) for mastectomy (fig 5, lower panel). 

Information was too limited on tumour related characteristics to conduct any meaningful analyses, with information missing on tumour size (72.7% of women), grade of invasive disease (62.2%), and oestrogen receptor status (88.3%). Only 1.4% were recorded as having oestrogen receptor positive status and receiving endocrine therapy (table S19). Information about margin status was not available.

## Discussion

### Principal findings

Women diagnosed with non-screen detected ductal carcinoma in situ in England had around a four times greater risk of invasive breast cancer and of breast cancer death than women in the general population. For both endpoints, the increased risks lasted until at least 25 years after diagnosis. For invasive breast cancer, the cumulative risks were highest for women aged <50 years or ≥71 years at diagnosis, that is, for women who would not be invited for screening by the population-based NHS breast screening programme. For breast cancer death, the risks were highest for women aged <45 years or >60 years. The cumulative risks of invasive breast cancer and breast cancer death for women with non-screen detected ductal carcinoma in situ were higher than for women with screen detected ductal carcinoma in situ, but the absolute differences at 25 years were small (1.6 percentage points for invasive breast cancer and 0.6 percentage points for breast cancer death). There is no evidence that invasive breast cancer rates are decreasing for women whose ductal carcinoma in situ was diagnosed more recently, but breast cancer death rates for women diagnosed more recently have decreased significantly, probably due to improved treatment of invasive disease.

In this observational study, breast cancer mortality rates were similar in the various treatment groups. However, women with unilateral ductal carcinoma in situ undergoing breast conserving surgery had substantially higher ipsilateral invasive breast cancer rates than those undergoing mastectomy. Among women undergoing breast conserving surgery, ipsilateral invasive breast cancer rates were highest in women who did not receive radiotherapy, but even among those who received radiotherapy, the 25 year cumulative incidence rate was more than double that for women undergoing mastectomy.

### Strengths and limitations

This study is the first, to our knowledge, to characterise the long term risks of invasive breast cancer and of breast cancer death in all women diagnosed with ductal carcinoma in situ outside the NHS breast screening programme, and it accompanies our previous study of women diagnosed within the NHS breast screening programme.^4^ We have considered invasive recurrences only and not recurrences of ductal carcinoma in situ because, while the national disease registration service has near complete follow-up for invasive breast cancer, breast cancer death, and deaths from other causes, no mechanisms are in place for complete identification of recurrence of ductal carcinoma in situ.

The data available had some limitations. Information about surgery and radiotherapy treatment was not recorded for 16.8% (4582/27 335) of women with unilateral non-screen detected ductal carcinoma in situ. Also, we did not have information on the route to diagnosis for women with non-screen detected ductal carcinoma in situ. They may have presented symptomatically (perhaps as an interval ductal carcinoma in situ diagnosed between NHS screening mammograms), had ductal carcinoma in situ diagnosed incidentally from imaging undertaken for other reasons, or had screening undertaken in the private sector. Incidental diagnosis is a likely reason for younger women who were not eligible for the NHS breast screening programme. For those of screening age, any of these reasons are possible, and one possible explanation for the increasing cumulative risk of invasive breast cancer with increasing calendar period in the non-screen detected group is an increase in the proportion of cases that were interval ductal carcinoma in situ, with more aggressive characteristics (fig 4). Whilst some of the lower rates of invasive breast cancer and breast cancer death in women with screen detected compared with non-screen detected ductal carcinoma in situ might be attributable to differences in lifestyle and health behaviour,^8^ the limited socioeconomic information that we had for these cohorts suggested that no large differences existed.

Data are missing regarding tumour related factors, so study of the associations between these characteristics and outcomes in non-screen detected ductal carcinoma in situ was not possible, neither was comparison of tumour characteristics in women in non-screen detected and screen detected ductal carcinoma in situ. Likewise, information about margin distance after surgical resection was not available, which meant that these data could not contribute further to the discussion on optimal margin distance. Nor did we have information on women for whom ductal carcinoma in situ was not their first cancer. Cause of death was missing for a small proportion of women, so that our estimated rates for breast cancer death may be slightly low. Despite these limitations, we consider the overall quality of the data underpinning the conclusions in our study remains high.

### Comparison with other studies

A previous study examined the risks of invasive breast cancer and breast cancer death solely in women with screen detected ductal carcinoma in situ in England.^4^ By studying women diagnosed outside the NHS breast screening programme and with long follow-up, this study allows for a complete picture of the long term risks of both invasive breast cancer and breast cancer death after diagnosis of ductal carcinoma in situ among women in England. Our findings also inform the discussion on whether the mode of detection should be used in the treatment decision making process as a prognostic consideration.^9^ Similar work in the Netherlands comparing invasive breast cancer after screen detected and non-screen detected ductal carcinoma in situ estimated the absolute difference in the risk of ipsilateral invasive breast cancer at 15 years to be 1.0 percentage point.^10^ Our work has found a similar difference at 15 years (1.4 percentage points) and also provided estimates of the differences at 25 years of 1.6 percentage points for invasive breast cancer and 0.6 percentage points for breast cancer death. While we had sparse information about oestrogen receptor status and endocrine therapy use, National Institute of Clinical Excellence (NICE) guidelines did not introduce a recommendation that clinicians discuss the use of endocrine treatment for women with ductal carcinoma in situ after breast conserving surgery until July 2018,^11^ and therefore few women in our study are likely to have received this treatment.

A previous study of 108 196 women with ductal carcinoma in situ in the United States has reported a cumulative risk of breast cancer mortality of 3.3% at 20 years after diagnosis of ductal carcinoma in situ.^12^ While that study could not separate women with screen detected and non-screen detected ductal carcinoma in situ, their reported risk was lower than the 20 year breast cancer death risk observed in our study for women with either non-screen detected ductal carcinoma in situ (6.1%) or screen detected ductal carcinoma in situ (4.4%). The screening interval is generally shorter in the United States and other high income countries than the three yearly intervals used in the UK. As a result, the proportion of non-screen detected ductal carcinoma in situ diagnoses that are interval cases (ie, arising in between screens) would be expected to be higher in the UK than in the United States or other high-income countries.

Ipsilateral invasive breast cancer rates were much lower following mastectomy than following breast conserving surgery, with or without radiotherapy, in the present study, in our previous work,^4^ and in a population based cohort of 10 090 women with ductal carcinoma in situ in the Netherlands.^13^ The consistency of these observational studies is notable. However, the overall benefits and risks of treatment can only be evaluated reliably in the setting of randomised trials with long term follow-up. An overview of randomised trials of radiotherapy versus not in ductal carcinoma in situ found that radiotherapy after breast conserving surgery reduced the absolute 10-year risk of any ipsilateral breast event by around 15% but, like the present study, found no significant reduction in breast cancer mortality.^14^ Several other trials comparing non-operative treatments in ductal carcinoma in situ are currently underway.^1^ ^2^ ^3^ ^15^

### Conclusion and policy implications

Surveillance of women after a diagnosis of ductal carcinoma in situ currently focuses on yearly mammograms for the first five years after diagnosis, with women who are then aged 50-70 years entering the NHS breast screening programme and receiving invitations to attend for screening mammography at three yearly intervals thereafter, until aged 70 years.^16^ We have, however, provided evidence that the increased risk of invasive disease and breast cancer death following a diagnosis of ductal carcinoma in situ in both screen detected and non-screen detected ductal carcinoma in situ lasts for at least 25 years. These findings should inform considerations regarding the frequency and duration of surveillance following a diagnosis of ductal carcinoma in situ, particularly for women diagnosed at younger ages. Our results also suggest that, although not affecting breast cancer mortality, women who receive a mastectomy have lower long term risks of invasive disease than those who receive breast conserving surgery, even when accompanied by radiotherapy—but this difference has yet to be confirmed in a randomised trial. Finally, the higher risks shown in this study for women aged below 50 years at diagnosis should help to inform discussions in the clinic on the optimal management of these patients.

**What is already known on this topic**

Ductal carcinoma in situ is usually diagnosed within the NHS breast screening programme, but some diagnoses occur outside the programme, both in women of screening age and in older and younger women
Following screen detected ductal carcinoma in situ, the rates of invasive breast cancer and of breast cancer death are more than double those of the general population
Rates of invasive breast cancer and breast cancer death after non-screen detected ductal carcinoma in situ are uncertain

**What this study adds**

Following non-screen detected ductal carcinoma in situ, rates of invasive breast cancer and breast cancer deaths were about four times more than those of the general population
Women with unilateral ductal carcinoma in situ undergoing mastectomy had a lower ipsilateral invasive breast cancer rate than those undergoing breast conserving surgery, but breast cancer mortality rates were similar
Increased risks of invasive breast cancer and of breast cancer death lasted for at least 25 years, suggesting that ductal carcinoma in situ survivors may benefit from surveillance for at least three decades

## Data Availability

De-personalised study data may be made available on request to accredited researchers who submit a proposal that is approved by NHS Digital’s Data Access Request Service.

## Web extra

Extra material supplied by authors

Web appendix: Statistical methods and results

## Notes

Contributors: GSM, ZW and SCD contributed equally to this paper. GSM and SCD contributed to the NINBE study conceptualisation and design. JB and JC contributed to data collection. ZW, GSM, DD, and SCD contributed to data analysis, interpretation, and manuscript preparation. GSM and DD did the literature search. All authors read and approved the final version. GSM is the overall study guarantor. The corresponding author attests that all listed authors meet authorship criteria and that no others meeting the criteria have been omitted.

The lead author (GM) affirms that this manuscript is an honest, accurate, and transparent account of the study being reported; that no important aspects of the study have been omitted; and that any discrepancies from the study as planned have been explained.

Dissemination to participants and related patient and public communities: The results have already been presented to representatives of appropriate patient groups. After publication they will be freely available both to stakeholders and to the broader public.

Provenance and peer review: Not commissioned; externally peer reviewed.
