# Supplementary material for: Invasive breast cancer and breast cancer death after non-screen detected ductal carcinoma in situ from 1990 to 2018 in England: population based cohort study
Source: BMJ. 2024 Jan 24;384:e075498. doi: 10.1136/bmj-2023-075498 (PMC10806881; doi:10.1136/bmj-2023-075498)
Supplement: Supplementary file 1 — Web appendix: Statistical methods and results [file mang075498.ww.pdf]

Invasive breast cancer and breast cancer mortality after non-screen-detected DCIS: cohort study of all 27,543 women diagnosed outside the NHS Breast Screening Programme in England from 1990 to 2018

Supplementary Material

Contents

Supplementary Text, Tables and Figures are listed in the order they are referred to in the paper.

Title	Page
**Supplementary Text S1**<br>Statistical Methods.	6
**Supplementary Table S1**<br>Distribution of 27,335 women in England diagnosed with unilateral non-screen-detected ductal carcinoma in situ (DCIS) during 1990-2018 according to whether or not type of treatment was missing and year of DCIS diagnosis, and whether or not type of treatment was missing and age at DCIS diagnosis.	12
**Supplementary Table S2**<br>Distribution of 27,543 women in England diagnosed with non-screen-detected ductal carcinoma in situ (DCIS) during 1990-2018 according to emigration status and year of DCIS diagnosis, and emigration status and age at DCIS diagnosis.	13
**Supplementary Table S3**<br>Mortality from causes other than breast cancer: Woman-years at risk and numbers of observed and expected deaths from causes other than breast cancer or from an unknown cause by age at DCIS diagnosis and years since DCIS diagnosis in 27,543 women with non-screen-detected DCIS during 1990-2018. Expected values are based on mortality rates for England and Wales.	14
**Supplementary Table S4**<br>Mortality from causes other than breast cancer: Numbers of observed and expected deaths from causes other than breast cancer or from an unknown cause by calendar period of DCIS diagnosis and years since DCIS diagnosis in 27,543 women with non-screen-detected DCIS during 1990-2018. Expected values are based on mortality rates for England and Wales.	14

Supplementary Table S5 15
Invasive breast cancers: Woman-years at risk and numbers of observed and expected invasive breast cancers (IBC) by age at DCIS diagnosis and years since DCIS diagnosis in 27,543 women with non-screen-detected DCIS during 1990-2018. Expected values are based on cancer incidence rates in England.

Supplementary Table S6 15
Invasive breast cancers: Numbers of observed and expected invasive breast cancers (IBC) by calendar period of DCIS diagnosis and years since DCIS diagnosis in 27,543 women with non-screen-detected DCIS during 1990-2018. Expected values are based on cancer incidence rates in England.

Supplementary Table S7 16
Breast cancer death: Woman-years at risk and numbers of observed and expected breast cancer deaths (BCD) by age at DCIS diagnosis and years since DCIS diagnosis in 27,543 women with non-screen-detected DCIS during 1990-2018. Expected values are based on mortality rates for England and Wales.

Supplementary Table S8 16
Breast cancer death: Numbers of observed and expected breast cancer deaths (BCD) by calendar period of DCIS diagnosis and years since DCIS diagnosis in 27,543 women with non-screen-detected DCIS during 1990-2018. Expected values are based on mortality rates for England and Wales.

Supplementary Table S9 17
Invasive breast cancer: Values used in figure 2 for cumulative risk of invasive breast cancer in 27,543 women with non-screen-detected DCIS by age at diagnosis of DCIS and time since diagnosis. Cumulative risks take into account competing risks from other causes of death. Expected values are based on cancer incidence rates for England and mortality rates for England and Wales.

Supplementary Table S10 19
Breast cancer death: Values used in figure 2 for cumulative risks of breast cancer death (BCD). Cumulative risk of invasive breast cancer and of death from breast cancer in 27,543 women with non-screen-detected DCIS by age at diagnosis of DCIS and time since diagnosis. Cumulative risks take into account competing risks

from other causes of death. Expected values are based on cancer incidence rates for England and mortality rates for England and Wales.

Supplementary Table S11 21
Invasive breast cancer: Values used in figure 3 for cumulative risk of invasive breast cancer in 27,543 women with non-screen-detected DCIS by year of diagnosis of DCIS and time since diagnosis. Cumulative risks take into account competing risks from other causes of death. Expected values are based on cancer incidence rates for England and mortality rates for England and Wales.

Supplementary Table S12 22
Breast cancer death: Values used in figure 3 for cumulative risks of breast cancer death (BCD). Cumulative risk of death from breast cancer in 27,543 women with non-screen-detected DCIS by year of diagnosis of DCIS and time since diagnosis. Cumulative risks take into account competing risks from other causes of death. Expected values are based on cancer incidence rates for England and mortality rates for England and Wales.

Supplementary Table S13 23
Characteristics of 9679 women with non-screen-detected unilateral ductal carcinoma in situ (DCIS) at ages 50-64 years, and 31,141 women with screen-detected unilateral DCIS at ages 50-64 DCIS, diagnosed in England during 1990-2018 and their status on December 2018.

Supplementary Table S14 24
Invasive breast cancer: Ratios of invasive breast cancer incidence rate in women with non-screen-detected DCIS compared with women with screen-detected DCIS. 9679 women aged 50-64 years with unilateral non-screen-detected ductal carcinoma in situ (DCIS) and 31,141 women with screen-detected DCIS in England.

Supplementary Table S15 24
Breast cancer death: Ratios of breast cancer death rate in women with non-screen-detected DCIS compared with women with screen-detected DCIS. 9679 women aged 50-64 years with non-screen-detected unilateral ductal carcinoma in situ (DCIS) and 31,141 women with screen-detected DCIS in England.

Supplementary Table S16 25
Values used in figure 4 for comparison of cumulative risks of invasive breast cancer between non-screen-detected and screen-detected DCIS in women aged 50-64 yrs, by year of diagnosis and time since diagnosis. Cumulative risks take into account competing risks from other causes of death.

Supplementary Table S17 26
Values used in figure 4 for comparison of cumulative risks of breast cancer death between non-screen-detected and screen-detected DCIS in women aged 50-64 yrs, by year of diagnosis and time since diagnosis. Cumulative risks take into account competing risks from other causes of death.

Supplementary Table S18 27
Values used in figure 5 for cumulative rates and 95% confidence intervals in 22,753 women with non-screen-detected unilateral DCIS during 1990-2018

Supplementary Table S19 28
The distributions of clinical variables by calendar period (censored on 30 Dec 2018).

Supplementary Text S1. Statistical Methods

A Tabulation of woman**-years at risk and observed events**

The analyses were conducted using the standard epidemiological approach of considering the numbers of observed events (eg invasive breast cancers, breast cancer deaths, or deaths from other causes) and the woman-years at risk (i.e. for each woman the total length of time during which she was at risk of experiencing an event). The methods described here are for invasive breast cancer (IBC) in women diagnosed with non-screen-detected ductal carcinoma in situ (DCIS). The methods for the other endpoints are similar, as are the methods for women with screen-detected DCIS.

For each woman who was eligible for the study, the length of time from 6 months after her DCIS diagnosis until the earliest of IBC diagnosis, death, emigration, the woman's 90th birthday or 31st December 2018 was calculated. These lengths of time were added together and formed the woman-years at risk. The number of women whose contribution to the woman-years was terminated by an IBC occurring was obtained in a similar fashion. For each analysis both the woman-years and the number of IBCs were then tabulated according to all the factors needed for that particular analysis.

B Significance tests for heterogeneity in proportion of women according to categories of age at diagnosis, year of diagnosis, etc. (tables 1, S1-S2, S13)

Significance tests of whether the distribution of women across categories of one factor varied across categories of another factor (e.g. whether the distribution of women across treatment categories varied by calendar year of diagnosis) were conducted using Pearson's chi-squared test.

C Observed and numbers of expected events, and ratios of observeds to expecteds (tables S3-S12, S16-S17)

Observed rates per 1000 woman-years at risk were calculated by tabulating the numbers of observed IBCs and the woman-years according to the categories of the factors of interest (age at DCIS diagnosis, years since DCIS diagnosis, calendar year of diagnosis, etc). Then, for each category, the number of observed IBCs was divided by the corresponding number of woman-years and multiplied by 1000. Where appropriate, the categories for age at DCIS diagnosis were chosen to be compatible with the age ranges of women invited for screening. The categories of time since diagnosis were chosen after tabulating the rates in finer time categories to ensure that no features of the data would be concealed by the categorisation. Confidence intervals for rates in the study population were calculated by assuming that the numbers of IBCs had a Poisson distribution with a parameter that was constant within each category for which the data were tabulated, and that the numbers of woman-years were fixed.

continued overleaf

Calculation of the numbers of IBCs expected in the study population was based on IBC incidence rates for women in the entire population of England which were provided by the National Disease Registration Service. The rates were provided in five-year groups of attained age for each individual calendar year from 1988-2018. The woman-years for the study population were then tabulated by these same categories (i.e. five-year groups of attained age within each individual calendar year) as well as any additional factors of interest for a specific analysis (e.g. time since DCIS diagnosis). Within each combination of all the tabulated factors, the total number of woman-years was then multiplied by the relevant attained-age and calendar year specific IBC incidence rate for England and Wales. This provided the number of IBCs expected for that combination of factors. The expected IBCs were then summed over all the factors that were not of direct interest in that analysis, (e.g. in **table S5** attained age, calendar year of DCIS diagnosis and either time since DCIS diagnosis or age at DCIS diagnosis) to provide the number of IBCs expected by age at diagnosis or time since diagnosis. For each category of age at diagnosis or time since diagnosis, the ratio of observed to expected IBCs (sometimes referred to as the Standardised Incidence Ratio) was then calculated by dividing the number of IBCs observed by the number expected. The confidence intervals for the ratios of observed to expected IBCs were calculated assuming that the number of IBCs observed had a Poisson distribution while the number expected was fixed.

Expected numbers of deaths from breast cancer and from causes other than breast cancer were calculated in the same way, based on mortality rates for the entire population of England and Wales and provided by the Office for National Statistics.

D **Significance tests for trend or heterogeneity in the ratios of observed to population-based expected events for different levels of a patient characteristic and for interactions between two characteristics (tables S3-S8)**

Significance tests for a trend in the ratio of observed to population-based expected IBCs across one of the patient characteristics (e.g. age at DCIS diagnosis in table S5) were conducted using Poisson regression. To do this the numbers of observed and the numbers of population-based expected IBCs were tabulated according to the characteristic using the categories shown in the table. The numbers of IBCs observed in each category were assumed to have a Poisson distribution and the number of population-based expected IBCs were assumed to be fixed. The following model was then fitted to the numbers of observed IBCs in the different categories using maximum likelihood:

$$\exp(y_i+\mu p_i+\beta x_i)$$

where y_j is the number of woman-years in the category, μ is an unknown constant common to all categories, p_i is the population-based expected number of events in the cell, x_i indicates the category and β is an unknown parameter representing the trend in the ratio of observed to population-based events across the categories.

continued overleaf

Significance tests for a trend in the rate ratios for the different categories of the factor (i.e. of $\beta = 0$) were then carried out using a likelihood ratio test.
For tests of heterogeneity across the different categories, the following model was fitted:

$$\exp(y_j+\mu p_i+\beta_1 x_1+\beta_2 x_2+ \ldots\beta_k x_k)$$

$x_1, x_2, \ldots, x_k$ are indicator variables denoting the different categories of the factor under study, β_1 was assumed to be 0, setting the first category of the factor as the baseline category, and $\beta_2, \ldots, \beta_k$ were unknown parameters. Significance tests for heterogeneity between the rate ratios (i.e. of $\beta_1 = \beta_2 = \beta_3 \ldots = \beta_k = 0$) were carried out using a likelihood ratio test. Significance tests for interactions between the different factors (e.g. age at DCIS diagnosis and time since diagnosis in table S6) were carried out by comparing models that included terms for the interaction between two variables with models that did not and were, once again, carried out using the likelihood ratio test.

E **Cumulative observed risk of IBC, accounting for competing risks from causes of death other than breast cancer (figures 2-4, tables S9-S12, S16-S17)**
To derive the cumulative risk that a woman in the study population would develop IBC at 1, 2, 3, …, 18, 19, 20 years after her diagnosis of DCIS, it is necessary to take into account the fact that she may, during this period, die from a cause other than breast cancer (or from an unknown cause) before any IBC has had time to develop, thereby reducing the risk that she will develop IBC to below that suggested just by consideration of the age-specific IBC rates. The calculation was carried out as follows.

First we divided the years since diagnosis of DCIS into time intervals. The first time interval was of length six months (to account for the fact that women entered the study six months after their diagnosis of DCIS) and subsequent time intervals had length one year. We denote the annual IBC rate (i.e. the observed number of IBCs divided by the woman-years) during the tth interval by $r_{ibc,t}$ and the annual mortality rate from competing causes of death (CCD) during the tth interval by $r_{ccd,t}$.

Based on the standard theory of Poisson processes, the probability that a woman who has survived up to the beginning of time interval t without developing IBC will develop IBC during interval t is:

$$p_{ibc,t} = 1-\exp[- r_{ibc,t} T_t] , \quad (1)$$

where T_t is the length of time interval t in years, while the probability that a woman who has survived up to the beginning of time interval t will die from a CCD during interval t is:

$$p_{ccd,t} = 1-\exp[- r_{ccd,t} T_t] ,$$

continued overleaf

and the probability that a woman who has survived up to the beginning of time interval t without developing IBC will either develop IBC or die from a CCD during interval t is:

$$p_{ibc+ccd,t} = 1\text{-}\exp[- (r_{ibc,t} + r_{ccd,t})T_t] ,$$

and the probability that she will experience neither event during interval t is $(1\text{-}p_{ibc+ccd,t})$. The probability that a woman will survive up to the end of interval (t-1) without developing IBC can then be calculated recursively by the formula:

$$Q_{ibc+ccd,t-1} = Q_{ibc+ccd,t-2} (1\text{-}p_{ibc+ccd,t-1}) , \quad (2)$$

where $Q_{ibc+ccd,0}$ is set to 1.

It then follows that, by combining (1) and (2) above, the probability that a woman will develop an IBC during interval t, accounting for the competing risk of dying previously from another cause is:

$$Q_{ibc+ccd,t-1} [1\text{-}\exp(- r_{ibc,t} T_t)]. \quad (3)$$

For each interval, t, the quantities in (3) can then be summed over previous time intervals to obtain the cumulative probability of developing an IBC by the end of interval t. This value can then be multiplied by 100 to provide the corresponding risk in terms of percent.

The variance of the cumulative observed risk of IBC was derived from the formulae above, together with the assumption that the observed numbers of deaths in each time interval had independent Poisson distributions and that the annual mortality rates from competing causes of death (CCD) were known precisely. Confidence intervals were based on the variance.

F Cumulative expected risk of IBC, accounting for competing risks from causes of death other than breast cancer (figures 2-3, tables S9-S12)

The calculation of the cumulative expected risk of IBC accounting for competing risks from causes of death other than breast cancer was conducted in a similar fashion to that for the observed risk derived above, except that rather than using the observed annual IBC rate (i.e. the observed number of IBCs divided by the woman-years) we used the expected rates (i.e. the expected number of IBCs divided by the woman-years) where the expected number of IBCs had been calculated as described above in the section ‘Calculation of numbers of IBCs expected’.

continued overleaf

G Comparability between results for women diagnosed with DCIS in different calendar years (figures 2-4, tables S9-S12, S16-S17)

Death rates in women in the general population have changed during the period included in this study. We wished to find a way of examining whether the cumulative risk of developing breast cancer following a diagnosis of DCIS had changed that took account of CCD but was not heavily influenced by changes in the age-specific CCD rates. Therefore, rather than using individual calendar-year specific values for the CCD rates, we instead used the 2018 death rates for all causes other than breast cancer for England and Wales to account for mortality from CCD throughout. This enabled us to be sure that any changes in the cumulative risks of IBC for women diagnosed with DCIS in different calendar years would be attributable to changes in the rate of development of IBC rather than changes in the rates of CCD over calendar time, whilst still taking account of deaths from competing causes.

H Ratios of observed rates in different groups of women (tables S14-S15)

The ratios of the observed rates in women with screen-detected DCIS compared with women with non-screen-detected DCIS (**tables S14-S15)** were studied using Poisson regression. To do this the numbers of events and the woman-years at risk were tabulated by both time since diagnosis of DCIS (factor i) and calendar year of DCIS (factor j). The numbers of events in each cell of the table were assumed to have a Poisson distribution and the numbers of events expected in the ijth cell of the table were assumed to be given by:

$$\exp(y_{ij}+\mu+v_i+\lambda_{j+}\gamma_{ij})$$

where y_{ij} is the number of woman-years in the cell, μ is the difference between the non-screen-detected and the screen-detected groups, and v_i and λ_j are unknown parameters denoting the different categories of the factors of interest and the γ_{ij} are additional parameters representing an interaction between the time since diagnosis and calendar year of diagnosis. Models of this form were fitted to the data by the method of maximum likelihood and a test of γ_{ij} = 0 across all categories of both factors was carried out using a likelihood ratio test.

continued overleaf

I Cumulative rates (figures 5, table S18)

To obtain cumulative rates, the woman-years and IBCs were tabulated simultaneously according to categories of time since DCIS diagnosis and of the factor of interest (e.g. for **figure 5 (upper panel)**: Breast-conserving surgery with radiotherapy, Breast conserving surgery without radiotherapy, and Mastectomy). For time since diagnosis of DCIS the cut-points used to define the categories were chosen after inspection of the observed rates in finer time categories. For level k of the factor of interest, the cumulative rate in per-cent up to time T is then:

$$100\Sigma(O_{ibc,t}\, d_t/y_t)$$

where $O_{ibc,t}$ is the number of observed events in time category t, y_t is the number of woman-years in category t, d_t is the length of category t in years, and summation is over all categories of t up to T. Checks were made that adjustment for the potential confounding factors (calendar year of exposure, age at exposure, time since exposure) made no material difference to the results. Confidence intervals for rates in the study population were calculated by assuming that the numbers of IBCs in each category had a Poisson distribution.

Supplementary Table S1 Distribution of 27,335 women in England diagnosed with unilateral non-screen-detected ductal carcinoma in situ (DCIS) during 1990-2018 according to whether or not type of treatment was missing and year of DCIS diagnosis, and whether or not type of treatment was missing and age at DCIS diagnosis.

Variable	Category	Treatment reported	Treatment unknown	Treatment missing (%)	P-val*	Total
Year of DCIS diagnosis	1990-99	5343 (23.5)	2598 (56.7)	32.7	<0.001	7941 (29.1)
	2000-09	8363 (36.8)	904 (19.7)	9.8		9267 (33.9)
	2010-18	9047 (39.8)	1080 (23.6)	10.7		10127 (37.0)
Age at DCIS diagnosis	<45	4662 (20.5)	577 (12.6)	11.0	<0.001	5239 (19.2)
	45-49	4073 (17.9)	514 (11.2)	11.2		4587 (16.8)
	50-59	5436 (23.9)	1417 (30.9)	20.7		6853 (25.1)
	60-70	4378 (19.2)	1019 (22.2)	18.9		5397 (19.7)
	71+	4204 (18.5)	1055 (23.0)	20.1		5259 (19.2)
Total	-	22753	4582	16.8	-	27335 (100.0)

*P-val for heterogeneity test

Supplementary Table S2 Distribution of 27,543 women in England diagnosed with non-screen-detected ductal carcinoma in situ (DCIS) during 1990-2018 according to emigration status and year of DCIS diagnosis, and emigration status and age at DCIS diagnosis.

Year of DCIS diagnosis	Followed	Emigrated	P-val	Total
1990-99	7,821 (97.85)	172 (2.15)	0.003	7,993 (100.00)
2000-09	9,164 (98.39)	150 (1.61)		9,314 (100.00)
2010-18	10,080 (98.48)	156 (1.52)		10,236 (100.00)
Total	27,065 (98.26)	478 (1.74)		27,543 (100.00)

Age at DCIS diagnosis	Followed	Emigrated	P-val	Total
<45	5,157 (97.80)	116 (2.20)	<0.001	5,273 (100.00)
45-49	4,547 (98.21)	83 (1.79)		4,630 (100.00)
50-59	6,770 (98.00)	138 (2.00)		6,908 (100.00)
60-70	5,345 (98.38)	88 (1.62)		5,433 (100.00)
71+	5,246 (99.00)	53 (1.00)		5,299 (100.00)
Total	27,065 (98.26)	478 (1.74)		27,543 (100.00)

*P-val for heterogeneity test

Supplementary Table S3. Mortality from causes other than breast cancer: Woman-**years at risk and numbers of observed and expected deaths from causes other than breast cancer or from an unknown cause by age at DCIS diagnosis and years since DCIS diagnosis in 27,543 women with non-screen-detected DCIS during 1990-2018. Expected values are based on mortality rates for England and Wales.**

	**Number of observed deaths**	**Woman-years at risk**	**Death rate per 1000 woman-years (95% CI)**	**Number of expected deaths**	**Ratio of observed to expected (95% CI)**
**Age at DCIS diagnosis (years)**					
<45	106	59,035	1.80 (1.48- 2.17)	136.7	0.78 (0.64-0.94)
45-49	167	50,737	3.29 (2.83- 3.83)	223.46	0.75 (0.64-0.87)
50-59	772	93,276	8.28 (7.71- 8.88)	982.47	0.79 (0.73-0.84)
60-70	1353	61,804	21.89 (20.76-23.09)	1765.42	0.77 (0.73-0.81)
71+	1632	32,566	50.11 (47.74-52.60)	2385.91	0.68 (0.65-0.72)
p for heterogeneity across 4 categories:			<0.001		0.01
p for trend across 4 categories:			<0.001		<0.001
**Time since DCIS diagnosis (years)**					
0.5-	138	13,539	10.19 (8.63-12.04)	186.65	0.74 (0.63-0.87)
1-	240	25,835	9.29 (8.19-10.54)	364.25	0.66 (0.58-0.75)
2-	238	24,222	9.83 (8.65-11.16)	351.19	0.68 (0.60-0.77)
3-	221	22,587	9.78 (8.58-11.16)	336.21	0.66 (0.58-0.75)
4-	249	21,010	11.85 (10.47-13.42)	324.97	0.77 (0.68-0.87)
0.5-4	1086	107,194	10.13 (9.55-10.75)	1563.27	0.69 (0.65-0.74)
5-9	1011	84,031	12.03 (11.31-12.80)	1417.58	0.71 (0.67-0.76)
10-19	1459	88,728	16.44 (15.62-17.31)	1898.56	0.77 (0.73-0.81)
20+	474	17,465	27.14 (24.80-29.70)	614.54	0.77 (0.70-0.84)
p for heterogeneity across 4 categories:			<0.001		0.04
p for trend across 4 categories:			<0.001		0.01
**Total**	4030	297,418	13.55 (13.14-13.97)	5493.96	0.73 (0.71-0.76)
P for test of observed/expected=1					<0.001

CI: confidence interval

Supplementary Table S4. Mortality from causes other than breast cancer: Numbers of observed and expected deaths from causes other than breast cancer or from an unknown cause by calendar period of DCIS diagnosis and years since DCIS diagnosis in 27,543 women with non-screen-detected DCIS during 1990-2018. Expected values are based on mortality rates for England and Wales.

**Time since DCIS diagnosis (years)**	**1990-1999**			**2000-2009**			**2010-2018**		
	**Obs. IBC**	**Exp. IBC**	**Ratio (95% CI)**	**Obs. IBC**	**Exp. IBC**	**Ratio (95% CI)**	**Obs. IBC**	**Exp. IBC**	**Ratio (95% CI)**
0.5-	24	47.48	0.51 (0.34-0.75)	56	68.17	0.82 (0.63-1.07)	58	71	0.82 (0.63-1.06)
1-	55	97.91	0.56 (0.43-0.73)	95	138.49	0.69 (0.56-0.84)	90	127.85	0.70 (0.57-0.87)
2-	61	102.09	0.60 (0.46-0.77)	99	138.63	0.71 (0.59-0.87)	78	110.48	0.71 (0.57-0.88)
3-	67	106.45	0.63 (0.50-0.80)	99	137.63	0.72 (0.59-0.88)	55	92.13	0.60 (0.46-0.78)
4-	88	108.9	0.81 (0.66-1.00)	95	141.42	0.67 (0.55-0.82)	66	74.65	0.88 (0.69-1.13)
p for trend with year of diagnosis within 5 year of diagnosis: 0.09 (unadjusted), 0.06 *(adjusted for time since diagnosis and age at diagnosis)*									
0.5-4	295	462.83	0.64 (0.57-0.71)	444	624.33	0.71 (0.65-0.78)	347	476.11	0.73 (0.66-0.81)
5-9	402	574.31	0.70 (0.63-0.77)	497	715.35	0.69 (0.64-0.76)	112	127.93	0.88 (0.73-1.05)
10-19	1006	1318.87	0.76 (0.72-0.81)	453	579.69	0.78 (0.71-0.86)			
20+	474	614.54	0.77 (0.70-0.84)						
**Total**	2177	2970.55	0.73 (0.70-0.76)	1394	1919.37	0.73 (0.69-0.77)	459	604.04	0.76 (0.69-0.83)
p for trend with year of diagnosis considering all years since diagnosis: 0.68 (unadjusted), 0.01 (adjusted for time since diagnosis and age at diagnosis)									
p for interaction between time since DCIS diagnosis and age at diagnosis: p=0.12									

CI: confidence interval

Supplementary Table S5. Invasive breast cancers: Woman**-years at risk and numbers of observed and expected invasive breast cancers (IBC) by age at DCIS diagnosis and years since DCIS diagnosis in 27,543 women with non-screen-detected DCIS during 1990-2018. Expected values are based on cancer incidence rates in England.**

	Number of observed IBCs	Womann-years at isk	Incidence rate per 1000 womann-years (95% CI)	Number of expected IBCs	Ratio of observed to expected (95% CI)
**Age at DCIS diagnosis (years)**					
<45	791	53436	14.80 (13.81-15.87)	107.87	7.33 (6.84-7.86)
45-49	581	47149	12.32 (11.36-13.37)	135.79	4.28 (3.94-4.64)
50-59	981	86,285	11.37 (10.68-12.10)	291.25	3.37 (3.16-3.59)
60-70	726	57,392	12.65 (11.76-13.60)	207.97	3.49 (3.25-3.75)
71+	572	30,739	18.61 (17.14-20.20)	125.01	4.58 (4.22-4.97)
P for heterogeneity:			<0.001		<0.001
P for trend:			0.02		<0.001
**Time since DCIS diagnosis (years)**					
0.5-	168	13,496	12.45 (10.70-14.48)	34.88	4.82 (4.14-5.60)
1-	398	25,505	15.60 (14.14-17.22)	67.65	5.88 (5.33-6.49)
2-	403	23,595	17.08 (15.49-18.83)	64.78	6.22 (5.64-6.86)
3-	332	21,731	15.28 (13.72-17.01)	61.82	5.37 (4.82-5.98)
4-	299	19,995	14.95 (13.35-16.75)	58.81	5.08 (4.54-5.69)
0.5-4	1600	104,322	15.34 (14.60-16.11)	287.94	5.56 (5.29-5.84)
5-9	1056	77,978	13.54 (12.75-14.38)	244.34	4.32 (4.07-4.59)
10-19	849	78,108	10.87 (10.16-11.63)	277.30	3.06 (2.86-3.27)
20+	146	14,593	10.00 (8.51-11.77)	58.30	2.50 (2.13-2.95)
P for heterogeneity across 4 categories:			<0.001		<0.001
P for trend across 4 categories:			<0.001		<0.001
**Total**	3651	275,001	13.28 (12.85-13.71)	867.88	4.21 (4.07-4.35)
P for test of observed/expected=1					<0.001

IBC: invasive breast cancer
CI: confidence interval

Supplementary Table S6. Invasive breast cancers: Numbers of observed and expected invasive breast cancers (IBC) by calendar period of DCIS diagnosis and years since DCIS diagnosis in 27,543 women with non-screen-detected DCIS during 1990-2018.
Expected values are based on cancer incidence rates in England.

Time since DCIS diagnosis (years)	1990-1999			2000-2009			2010-2018		
	Obs. IBC	Exp. IBC	Ratio (95% CI)	Obs. IBC	Exp. IBC	Ratio (95% CI)	Obs. IBC	Exp. IBC	Ratio (95% CI)
0.5-	35	8.82	3.97 (2.85-5.53)	58	12.15	4.77 (3.69-6.17)	75	13.91	5.39 (4.30-6.76)
1-	109	18.33	5.95 (4.93-7.17)	132	24.39	5.41 (4.56-6.42)	157	24.93	6.30 (5.39-7.36)
2-	122	19.10	6.39 (5.35-7.63)	147	24.47	6.01 (5.11-7.06)	134	21.21	6.32 (5.33-7.48)
3-	94	19.94	4.71 (3.85-5.77)	143	24.46	5.85 (4.96-6.89)	95	17.42	5.45 (4.46-6.67)
4-	111	20.63	5.38 (4.47-6.48)	104	24.58	4.23 (3.49-5.13)	84	13.60	6.18 (4.99-7.65)
P for trend with year of diagnosis within 5 year of diagnosis: 0.11 (unadjusted), 0.95 *(adjusted for time since diagnosis and age at diagnosis)*									
0.5-4	471	86.82	5.43 (4.96-5.94)	584	110.06	5.31 (4.89-5.75)	545	91.06	5.98 (5.50-6.51)
5-9	452	103.73	4.36 (3.97-4.78)	500	118.80	4.21 (3.86-4.59)	104	21.81	4.77 (3.94-5.78)
10-19	583	191.84	3.04 (2.80-3.30)	266	85.46	3.11 (2.76-3.51)			
20+	146	58.30	2.50 (2.13-2.95)						
**Total**	1652	440.68	3.75 (3.57-3.93)	1350	314.33	4.29 (4.07-4.53)	649	112.87	5.75 (5.32-6.21)
P for trend with year of diagnosis considering all years since diagnosis: 0.006(unadjusted), 0.45 (adjusted for time since diagnosis and age at diagnosis)									
P for interaction between time since DCIS diagnosis and age at diagnosis: p=0.69									

CI: confidence interval

Supplementary Table S7. Breast cancer death: Woman**-years at risk and numbers of observed and expected breast cancer deaths (BCD) by age at DCIS diagnosis and years since DCIS diagnosis in 27,543 women with non-screen-detected DCIS during 1990-2018. Expected values are based on mortality rates for England and Wales.**

	Number of observed BCDs	Womann-years at risk	BCD rate per 1000 womann-years (95% CI)	Number of expected BCDs	Ratio of observed to expected (95% CI)
**Age at DCIS diagnosis (years)**					
<45	176	59,035	2.98 (2.57-3.46)	17.18	10.25 (8.84-11.88)
45-49	118	50,737	2.33 (1.94-2.79)	23.63	4.99 (4.17- 5.98)
50-59	232	93,276	2.49 (2.19-2.83)	67.35	3.44 (3.03-3.92)
60-70	183	61,804	2.96 (2.56-3.42)	69.46	2.63 (2.28-3.05)
71+	199	32,566	6.11 (5.32-7.02)	59.54	3.34 (2.91-3.84)
P for heterogeneity:			<0.001		<0.001
P for trend:			<0.001		<0.001
**Time since DCIS diagnosis (years)**					
0.5-	15	13,539	1.11 (0.67-1.84)	9.17	1.64 (0.99-2.71)
1-	55	25,835	2.13 (1.63-2.77)	17.74	3.10 (2.38-4.04)
2-	57	24,222	2.35 (1.82-3.05)	16.93	3.37 (2.60-4.36)
3-	69	22,587	3.06 (2.41-3.87)	16.07	4.29 (3.39-5.43)
4-	74	21,010	3.52 (2.80-4.42)	15.28	4.84 (3.86-6.08)
0.5-4	270	107,194	2.52 (2.24-2.84)	75.19	3.59 (3.19-4.05)
5-9	304	84,031	3.62 (3.23-4.05)	64.37	4.72 (4.22-5.28)
10-19	268	88,728	3.02 (2.68-3.40)	77.84	3.44 (3.05-3.88)
20+	66	17,465	3.78 (2.97-4.81)	19.75	3.34 (2.63-4.25)
P for heterogeneity across 4 categories:			<0.001		<0.001
P for trend across 4 categories::			0.005		0.28
Total	908	297,418	3.05 (2.86-3.26)	237.15	3.83 (3.59-4.09)
P for test of observed/expected=1					<0.001

BCD: breast cancer death
CI: confidence interval

Supplementary Table S8. Breast cancer death: Numbers of observed and expected breast cancer deaths (BCD) by calendar period of DCIS diagnosis and years since DCIS diagnosis in 27,543 women with non-screen-detected DCIS during 1990-2018. Expected values are based on mortality rates for England and Wales.

Time since DCIS diagnosis (years)	1990-1999			2000-2009			2010-2018		
	Obs. IBC	Exp. IBC	Ratio (95% CI)	Obs. IBC	Exp. IBC	Ratio (95% CI)	Obs. IBC	Exp. IBC	Ratio (95% CI)
0.5-	4	3.14	1.28 (0.48-3.40)	5	3.13	1.60 (0.67-3.84)	6	2.91	2.07 (0.93-4.60)
1-	26	6.26	4.16 (2.83-6.10)	18	6.25	2.88 (1.81-4.57)	11	5.23	2.10 (1.17-3.80)
2-	21	6.23	3.37 (2.20-5.17)	27	6.21	4.35 (2.98-6.34)	9	4.49	2.01 (1.04-3.85)
3-	31	6.22	4.98 (3.50-7.09)	25	6.13	4.08 (2.76-6.04)	13	3.73	3.49 (2.03-6.01)
4-	30	6.17	4.86 (3.40-6.95)	31	6.14	5.05 (3.55-7.18)	13	2.97	4.37 (2.54-7.53)
P for trend with year of diagnosis within 5 year of diagnosis: 0.02 (unadjusted), 0.003 *(adjusted for time since diagnosis and age at diagnosis)*									
0.5-4	112	28.02	4.00 (3.32-4.81)	106	27.86	3.81 (3.15-4.60)	52	19.32	2.69 (2.05-3.53)
5-9	159	30.06	5.29 (4.53-6.18)	126	29.39	4.29 (3.60-5.11)	19	4.92	3.86 (2.46-6.05)
10-19	203	56.10	3.62 (3.15-4.15)	65	21.74	2.99 (2.35-3.81)			
20+	66	19.75	3.34 (2.63-4.25)						
**Total**	540	133.93	4.03 (3.71-4.39)	297	78.98	3.76 (3.36-4.21)	71	24.24	2.93 (2.32-3.70)
P for trend with year of diagnosis considering all years since diagnosis: 0.01 (unadjusted), <0.001 *(adjusted for time since diagnosis and age at diagnosis)*									
P for interaction between time since DCIS diagnosis and age at diagnosis: p=0.81									

CI: confidence interval

Supplementary Table S9. Invasive breast cancer: Values used in figure 2 for cumulative risk of invasive breast cancer in 27,543 women with non-screen-detected DCIS by age at diagnosis of DCIS and time since diagnosis. Cumulative risks take into account competing risks from other causes of death. Expected values are based on cancer incidence rates for England and mortality rates for England and Wales.

Time since DCIS diagnosis (years)	Number of woman-years	Number of IBC observed	Cumulative observed risk*	95% C.I. for cumulative observed risk*	Number of IBC expected*	Cumulative expected risk*
<45 years						
0.5-	2590	34	0.7	0.4- 0.9	2.7	0.1
1-	4921	88	2.4	2.0- 2.8	5.7	0.2
2-	4566	90	4.3	3.7- 4.9	6.0	0.3
3-	4232	71	5.9	5.2- 6.6	6.2	0.4
4-	3913	83	7.9	7.1- 8.6	6.2	0.6
5-	3614	57	9.3	8.5-10.2	6.3	0.8
6-	3345	53	10.7	9.8-11.6	6.4	1.0
7-	3083	46	12.0	11.1-13.0	6.4	1.2
8-	2841	47	13.5	12.4-14.5	6.3	1.4
9-	2596	27	14.4	13.3-15.4	6.0	1.6
10-	2358	30	15.4	14.3-16.6	5.7	1.8
11-	2122	27	16.5	15.3-17.7	5.3	2.1
12-	1907	13	17.0	15.8-18.3	4.9	2.3
13-	1717	19	17.9	16.7-19.2	4.5	2.6
14-	1532	17	18.8	17.5-20.1	4.1	2.8
15-	1356	13	19.6	18.2-20.9	3.7	3.1
16-	1196	19	20.8	19.3-22.3	3.4	3.4
17-	1034	11	21.6	20.1-23.1	3.0	3.6
18-	904	6	22.1	20.5-23.7	2.8	3.9
19-	780	10	23.1	21.4-24.7	2.4	4.2
20-	672	2	23.3	21.6-25.0	2.2	4.5
21-	567	7	24.2	22.4-26.0	1.9	4.8
22-	467	6	25.1	23.2-27.0	1.6	5.1
23-	368	4	25.9	23.8-27.9	1.3	5.4
24-	285	6	27.3	25.0-29.6	1.0	5.8†
45-49 years						
0.5-	2278	17	0.4	0.2- 0.5	4.8	0.1
1-	4339	66	1.9	1.5- 2.3	9.7	0.3
2-	4045	57	3.2	2.7- 3.8	9.7	0.6
3-	3752	50	4.5	3.9- 5.1	9.6	0.8
4-	3484	31	5.4	4.7- 6.1	9.4	1.1
5-	3211	46	6.7	5.9- 7.5	9.0	1.4
6-	2945	39	7.9	7.1- 8.8	8.3	1.6
7-	2701	29	8.9	8.0- 9.8	7.6	1.9
8-	2486	35	10.1	9.1-11.1	7.1	2.2
9-	2278	28	11.2	10.1-12.3	6.5	2.5
10-	2066	31	12.5	11.4-13.6	6.0	2.7
11-	1864	17	13.3	12.1-14.5	5.7	3.0
12-	1682	23	14.4	13.2-15.7	5.3	3.3
13-	1501	13	15.1	13.8-16.4	5.0	3.6
14-	1338	20	16.3	14.9-17.7	4.6	3.9
15-	1193	8	16.9	15.4-18.3	4.2	4.3
16-	1070	8	17.5	16.0-18.9	3.9	4.6
17-	954	10	18.3	16.7-19.8	3.6	4.9
18-	837	11	19.3	17.6-20.9	3.3	5.3
19-	710	10	20.3	18.6-22.0	2.9	5.6
20-	592	5	20.9	19.1-22.7	2.4	6.0
21-	496	7	21.9	20.0-23.9	2.0	6.3
22-	407	5	22.8	20.7-24.8	1.6	6.7
23-	320	6	24.1	21.8-26.3	1.2	7.0
24-	239	4	25.2	22.7-27.6	0.9	7.3†
50-59 years						
0.5-	3400	34	0.5	0.3- 0.7	9.1	0.1
1-	6523	73	1.6	1.3- 1.9	17.9	0.4
2-	6187	82	2.9	2.5- 3.3	17.6	0.7
3-	5815	79	4.2	3.7- 4.7	17.2	1.0
4-	5470	68	5.4	4.8- 5.9	16.8	1.3
5-	5139	75	6.7	6.1- 7.3	16.2	1.6
6-	4873	52	7.7	7.0- 8.4	15.7	1.9
7-	4596	59	8.8	8.1- 9.6	15.2	2.2
8-	4358	50	9.8	9.1-10.6	14.9	2.5
9-	4118	48	10.8	10.0-11.7	14.6	2.9
10-	3881	46	11.8	11.0-12.7	14.1	3.2

Table S9 continued on the next page.

Table S9 continued

Time since DCIS diagnosis (years)	Number of woman-years	Number of IBC observed	Cumulative observed risk*	95% C.I. for cumulative observed risk*	Number of IBC expected*	Cumulative expected risk*
50-59 years						
11-	3653	41	12.8	11.9-13.7	13.4	3.5
12-	3443	40	13.7	12.8-14.7	12.7	3.9
13-	3226	39	14.7	13.7-15.7	12.0	4.2
14-	3014	31	15.5	14.5-16.5	11.4	4.5
15-	2792	23	16.1	15.1-17.1	10.6	4.9
16-	2584	20	16.7	15.7-17.8	9.8	5.2
17-	2375	30	17.6	16.5-18.7	9.0	5.5
18-	2178	22	18.4	17.2-19.5	8.3	5.8
19-	1918	12	18.8	17.7-19.9	7.3	6.2
20-	1626	10	19.2	18.1-20.4	6.3	6.5
21-	1354	14	19.9	18.7-21.1	5.3	6.8
22-	1135	11	20.5	19.3-21.8	4.6	7.1
23-	919	9	21.1	19.8-22.4	3.8	7.4
24-	701	7	21.7	20.3-23.1	2.9	7.7†
60-70 years						
0.5-	2673	36	0.7	0.5- 0.9	8.6	0.2
1-	5109	57	1.8	1.4- 2.1	16.7	0.5
2-	4782	77	3.3	2.8- 3.8	15.8	0.8
3-	4467	69	4.8	4.2- 5.4	15.0	1.1
4-	4137	61	6.1	5.4- 6.8	14.2	1.5
5-	3844	40	7.0	6.3- 7.8	13.5	1.8
6-	3554	41	8.1	7.3- 8.8	12.6	2.1
7-	3281	47	9.3	8.4-10.1	11.7	2.4
8-	3042	48	10.6	9.7-11.5	10.9	2.8
9-	2779	45	11.9	10.9-12.8	9.9	3.1
10-	2575	32	12.8	11.8-13.8	9.3	3.4
11-	2363	21	13.5	12.4-14.5	8.7	3.7
12-	2153	15	14.0	12.9-15.0	8.1	4.0
13-	1952	17	14.6	13.5-15.7	7.6	4.3
14-	1773	21	15.4	14.2-16.5	7.1	4.6
15-	1600	16	16.0	14.8-17.2	6.5	4.9
16-	1423	24	17.0	15.8-18.3	5.9	5.2
17-	1249	10	17.5	16.2-18.7	5.3	5.5
18-	1094	13	18.1	16.8-19.4	4.7	5.7
19-	932	9	18.6	17.3-19.9	4.1	6.0
20-	730	8	19.1	17.7-20.4	3.3	6.2
21-	580	5	19.5	18.1-20.8	2.6	6.5
22-	451	5	19.9	18.5-21.3	2.1	6.7
23-	334	3	20.2	18.7-21.6	1.5	6.8
24-	244	5	20.8	19.3-22.3	1.1	7.0†
71+ years						
0.5-	2555	47	0.9	0.7- 1.2	9.6	0.2
1-	4613	114	3.3	2.8- 3.8	17.6	0.6
2-	4015	97	5.4	4.8- 6.1	15.7	0.9
3-	3465	63	6.9	6.2- 7.7	13.8	1.3
4-	2991	56	8.4	7.6- 9.2	12.2	1.6
5-	2542	49	9.7	8.9-10.6	10.5	1.9
6-	2140	34	10.8	9.8-11.7	8.9	2.2
7-	1817	19	11.4	10.4-12.4	7.7	2.5
8-	1524	28	12.4	11.4-13.5	6.5	2.8
9-	1270	14	13.0	11.9-14.1	5.5	3.0
10-	1057	12	13.5	12.4-14.6	4.6	3.3
11-	839	14	14.2	13.0-15.3	3.7	3.5
12-	637	11	14.8	13.6-16.0	2.8	3.7
13-	467	10	15.5	14.2-16.7	2.1	3.8
14-	328	4	15.8	14.5-17.1	1.5	4.0†

CI: confidence interval

*Risks given for the end of each period, i.e. risks in the line ‘9-‘ refer to 10 years after diagnosis, etc

†Risks at 25 and 15 years after diagnosis of DCIS respectively

Supplementary Table S10. Breast cancer death: Values used in figure 2 for cumulative risks of breast cancer death (BCD). Cumulative risk of invasive breast cancer and of death from breast cancer in 27,543 women with non-screen-detected DCIS by age at diagnosis of DCIS and time since diagnosis. Cumulative risks take into account competing risks from other causes of death. Expected values are based on cancer incidence rates for England and mortality rates for England and Wales.

Time since DCIS diagnosis (years)	Number of woman-years	Number of BCD observed	Cumulative observed risk*	95% CI for cumulative observed risk*	Number of BCD expected*	Cumulative expected risk*
<45 years						
0.5-	2598	0	0	0.0- 0.0	0.4	0
1-	4996	5	0.1	0.0- 0.2	0.8	0
2-	4716	8	0.3	0.1- 0.4	0.9	0
3-	4445	14	0.6	0.4- 0.8	0.9	0.1
4-	4170	21	1.1	0.8- 1.4	0.9	0.1
5-	3911	8	1.3	0.9- 1.6	0.9	0.1
6-	3666	16	1.7	1.3- 2.1	0.9	0.1
7-	3411	14	2.1	1.7- 2.6	0.9	0.2
8-	3177	10	2.4	1.9- 2.9	0.9	0.2
9-	2939	10	2.7	2.2- 3.3	0.9	0.2
10-	2692	8	3.0	2.5- 3.6	0.9	0.3
11-	2449	8	3.3	2.7- 3.9	0.8	0.3
12-	2210	8	3.7	3.0- 4.3	0.8	0.3
13-	1995	10	4.2	3.5- 4.9	0.8	0.4
14-	1801	3	4.3	3.6- 5.0	0.7	0.4
15-	1605	2	4.4	3.7- 5.2	0.7	0.4
16-	1422	3	4.6	3.9- 5.4	0.6	0.5
17-	1243	5	5.0	4.2- 5.8	0.6	0.5
18-	1095	3	5.2	4.4- 6.1	0.5	0.6
19-	952	3	5.5	4.6- 6.5	0.4	0.6
20-	820	2	5.8	4.8- 6.7	0.4	0.7
21-	693	6	6.5	5.4- 7.7	0.3	0.7
22-	577	3	7.0	5.7- 8.2	0.3	0.8
23-	464	2	7.4	6.0- 8.7	0.2	0.8
24-	370	1	7.6	6.2- 9.0	0.2	0.9†
45-49 years						
0.5-	2283	0	0	0.0-0.0	0.7	0
1-	4389	6	0.1	0.0-0.2	1.5	0
2-	4139	8	0.3	0.2-0.5	1.5	0.1
3-	3884	6	0.5	0.3-0.7	1.5	0.1
4-	3629	11	0.8	0.5-1.1	1.5	0.2
5-	3369	8	1.0	0.7-1.3	1.4	0.2
6-	3126	7	1.2	0.9-1.6	1.4	0.2
7-	2892	8	1.5	1.1-1.9	1.3	0.3
8-	2685	4	1.6	1.2-2.1	1.3	0.3
9-	2488	7	1.9	1.4-2.4	1.2	0.4
10-	2276	8	2.3	1.7-2.8	1.1	0.4
11-	2073	8	2.6	2.0-3.2	1.1	0.5
12-	1881	7	3.0	2.3-3.6	1.0	0.5
13-	1681	2	3.1	2.4-3.7	0.9	0.6
14-	1516	4	3.3	2.6-4.0	0.9	0.6
15-	1362	6	3.7	3.0-4.5	0.8	0.7
16-	1218	6	4.2	3.3-5.0	0.7	0.8
17-	1097	2	4.3	3.5-5.2	0.7	0.8
18-	974	0	4.3	3.5-5.2	0.6	0.9
19-	841	1	4.5	3.6-5.3	0.5	0.9
20-	712	5	5.1	4.0-6.1	0.5	1.0
21-	596	2	5.4	4.3-6.5	0.4	1.1
22-	488	0	5.4	4.3-6.5	0.4	1.1
23-	391	2	5.8	4.5-7.0	0.3	1.2
24-	294	0	5.8	4.5-7.0	0.2	1.2†
50-59 years						
0.5-	3409	2	0	0.0-0.1	1.9	0
1-	6592	7	0.1	0.0-0.2	3.7	0.1
2-	6316	8	0.3	0.1-0.4	3.7	0.1
3-	5998	13	0.5	0.3-0.6	3.6	0.2
4-	5704	14	0.7	0.5-0.9	3.5	0.3
5-	5405	22	1.1	0.8-1.4	3.4	0.3
6-	5155	21	1.5	1.2-1.8	3.3	0.4
7-	4903	18	1.9	1.5-2.2	3.2	0.5
8-	4673	9	2.0	1.7-2.4	3.1	0.5
9-	4457	12	2.3	1.9-2.7	3.1	0.6
10-	4234	9	2.5	2.1-2.9	3.0	0.6

Table S10 continued on the next page.

Table S10 continued

Time since DCIS diagnosis (years)	Number of woman-years	Number of BCD observed	Cumulative observed risk*	95% CI for cumulative observed risk*	Number of BCD expected*	Cumulative expected risk*
50-59 years						
11-	4020	11	2.7	2.3-3.2	2.9	0.7
12-	3815	12	3.0	2.6-3.5	2.8	0.8
13-	3608	8	3.2	2.7-3.7	2.7	0.9
14-	3409	7	3.4	2.9-3.9	2.6	0.9
15-	3178	8	3.6	3.1-4.2	2.5	1.0
16-	2955	10	3.9	3.4-4.5	2.4	1.1
17-	2741	5	4.1	3.5-4.7	2.3	1.1
18-	2526	5	4.3	3.7-4.8	2.3	1.2
19-	2229	6	4.5	3.9-5.1	2.1	1.3
20-	1898	7	4.8	4.1-5.4	1.9	1.4
21-	1586	2	4.9	4.2-5.5	1.7	1.5
22-	1338	5	5.2	4.5-5.9	1.5	1.6
23-	1092	5	5.5	4.7-6.3	1.3	1.7
24-	835	5	5.9	5.1-6.8	1.1	1.7†
60-70 years						
0.5-	2683	1	0	0.0-0.1	2.2	0
1-	5168	7	0.2	0.0-0.3	4.3	0.1
2-	4897	11	0.4	0.2-0.5	4.2	0.2
3-	4624	13	0.6	0.4-0.9	4.0	0.3
4-	4336	7	0.8	0.6-1.1	3.9	0.4
5-	4061	13	1.1	0.8-1.4	3.8	0.5
6-	3773	9	1.3	1.0-1.7	3.6	0.6
7-	3520	11	1.6	1.2-2.0	3.5	0.7
8-	3292	19	2.1	1.7-2.6	3.4	0.7
9-	3050	12	2.5	2.0-3.0	3.3	0.8
10-	2847	3	2.6	2.1-3.1	3.2	0.9
11-	2636	10	2.9	2.4-3.4	3.1	1.0
12-	2410	11	3.3	2.7-3.8	3.0	1.1
13-	2193	6	3.5	2.9-4.1	2.8	1.2
14-	2002	8	3.8	3.2-4.4	2.7	1.4
15-	1817	6	4.0	3.4-4.7	2.7	1.5
16-	1630	6	4.3	3.6-5.0	2.5	1.6
17-	1447	4	4.5	3.8-5.2	2.4	1.7
18-	1282	6	4.8	4.0-5.5	2.2	1.8
19-	1094	5	5.0	4.3-5.8	2.0	1.9
20-	863	3	5.2	4.4-6.0	1.7	2.0
21-	680	4	5.5	4.7-6.4	1.4	2.1
22-	528	1	5.6	4.7-6.5	1.1	2.2
23-	386	4	6.0	5.1-7.0	0.9	2.3
24-	278	1	6.2	5.2-7.1	0.7	2.4†
71+ years						
0.5-	2565	12	0.2	0.1-0.4	3.9	0.1
1-	4690	30	0.9	0.6-1.1	7.4	0.2
2-	4155	22	1.3	1.0-1.7	6.7	0.4
3-	3637	23	1.9	1.5-2.3	6.1	0.5
4-	3172	21	2.4	2.0-2.9	5.5	0.7
5-	2727	17	2.9	2.4-3.4	4.9	0.8
6-	2320	20	3.5	3.0-4.1	4.3	1.0
7-	1968	14	4.0	3.4-4.6	3.8	1.1
8-	1664	8	4.3	3.6-4.9	3.4	1.2
9-	1399	7	4.6	3.9-5.2	3.0	1.3
10-	1166	11	5.0	4.3-5.8	2.6	1.5
11-	933	3	5.2	4.5-5.9	2.2	1.6
12-	718	4	5.4	4.6-6.2	1.8	1.7
13-	535	3	5.6	4.8-6.4	1.4	1.8
14-	378	3	5.9	5.0-6.7	1.0	1.9†

CI: confidence interval
*Risks given for the end of each period, i.e. risks in the line ‘9-‘ refer to 10 years after diagnosis, etc
†Risks at 25 and 15 years after diagnosis of DCIS respectively

Supplementary Table S11. Invasive breast cancer: Values used in figure 3 for cumulative risk of invasive breast cancer in 27,543 women with non-screen-detected DCIS by year of diagnosis of DCIS and time since diagnosis. Cumulative risks take into account competing risks from other causes of death. Expected values are based on cancer incidence rates for England and mortality rates for England and Wales.

Time since DCIS diagnosis (years)	Number of woman-years	Number of IBC observed	Cumulative observed risk*	95% CI for cumulative observed risk*	Number of IBC expected*	Cumulative expected risk*
1990-1999						
0.5-	3981	35	0.4	0.3- 0.6	8.8	0.1
1-	7836	109	1.8	1.5- 2.1	18.3	0.3
2-	7654	122	3.3	2.9- 3.7	19.1	0.6
3-	7475	94	4.5	4.1- 5.0	19.9	0.8
4-	7284	111	5.9	5.4- 6.4	20.6	1.1
5-	7083	104	7.2	6.7- 7.8	20.9	1.4
6-	6898	86	8.3	7.7- 8.9	20.9	1.7
7-	6712	81	9.4	8.7-10.0	20.8	2.0
8-	6543	99	10.6	9.9-11.3	20.7	2.3
9-	6349	82	11.7	11.0-12.4	20.5	2.5
10-	6191	82	12.7	12.0-13.4	20.4	2.8
11-	6005	64	13.5	12.8-14.3	20.2	3.1
12-	5841	62	14.3	13.6-15.1	20.0	3.4
13-	5660	62	15.1	14.3-15.9	19.8	3.7
14-	5468	68	16.0	15.2-16.8	19.6	4.0
15-	5295	46	16.6	15.8-17.4	19.3	4.3
16-	5130	58	17.4	16.5-18.2	19.0	4.6
17-	4958	52	18.0	17.2-18.9	18.6	4.9
18-	4786	48	18.7	17.8-19.5	18.2	5.1
19-	4344	41	19.2	18.4-20.1	16.7	5.4
20-	3620	25	19.6	18.8-20.5	14.1	5.7
21-	2998	33	20.3	19.4-21.2	11.8	6.0
22-	2459	27	20.9	19.9-21.8	9.9	6.2
23-	1941	22	21.5	20.5-22.4	7.9	6.5
24-	1470	22	22.2	21.2-23.2	6.0	6.7†
2000-2009						
0.5-	4624	58	0.6	0.5- 0.8	12.2	0.1
1-	9059	132	2.1	1.8- 2.3	24.4	0.4
2-	8790	147	3.6	3.3- 4.0	24.5	0.7
3-	8507	143	5.2	4.8- 5.7	24.5	0.9
4-	8274	104	6.3	5.8- 6.8	24.6	1.2
5-	8043	112	7.6	7.0- 8.1	24.5	1.5
6-	7802	106	8.7	8.1- 9.3	24.3	1.8
7-	7566	101	9.8	9.2-10.4	24.1	2.1
8-	7335	101	10.9	10.3-11.6	23.8	2.4
9-	6693	80	11.8	11.2-12.5	22.1	2.6
10-	5747	69	12.8	12.1-13.4	19.3	2.9
11-	4836	56	13.6	12.9-14.3	16.6	3.2
12-	3982	40	14.3	13.6-15.1	13.9	3.5
13-	3203	36	15.1	14.3-15.9	11.3	3.8
14-	2517	25	15.8	14.9-16.6	9.0	4.1†
2010-2018						
0.5-	4891	75	0.8	0.6-0.9	13.9	0.1
1-	8611	157	2.5	2.2-2.9	24.9	0.4
2-	7150	134	4.3	3.9-4.8	21.2	0.7
3-	5749	95	5.8	5.3-6.4	17.4	1.0
4-	4436	84	7.5	6.9-8.1	13.6	1.3†

CI: confidence interval
*Risks given for the end of each period, i.e. risks in the line '9-' refer to 10 years after diagnosis, etc
†Risks at 25, 15, and 5 years after diagnosis of DCIS respectively

Supplementary Table S12. Breast cancer death: Values used in figure 3 for cumulative risks of breast cancer death (BCD). Cumulative risk of death from breast cancer in 27,543 women with non-screen-detected DCIS by year of diagnosis of DCIS and time since diagnosis. Cumulative risks take into account competing risks from other causes of death. Expected values are based on cancer incidence rates for England and mortality rates for England and Wales.

Time since DCIS diagnosis (years)	Number of woman-years	Number of BCD observed	Cumulative observed risk*	95% CI for cumulative observed risk*	Number of BCD expected*	Cumulative expected risk*
1990-1999						
0.5-	3989	4	0.1	0.0-0.1	3.1	0
1-	7914	26	0.4	0.2-0.5	6.3	0.1
2-	7818	21	0.6	0.5-0.8	6.2	0.2
3-	7716	31	1.0	0.8-1.3	6.2	0.3
4-	7600	30	1.5	1.1-1.7	6.2	0.4
5-	7463	36	1.9	1.6-2.2	6.1	0.4
6-	7326	33	2.3	2.0-2.6	6.0	0.5
7-	7195	31	2.7	2.3-3.0	6.0	0.6
8-	7071	29	3.0	2.7-3.4	6.0	0.7
9-	6939	30	3.5	3.0-3.8	6.0	0.8
10-	6825	21	3.7	3.3-4.1	6.0	0.8
11-	6676	28	4.1	3.6-4.5	5.9	0.9
12-	6534	32	4.5	4.0-4.9	5.8	1.0
13-	6373	20	4.7	4.3-5.2	5.7	1.1
14-	6215	19	5.0	4.5-5.5	5.6	1.2
15-	6062	18	5.2	4.7-5.7	5.5	1.2
16-	5902	23	5.5	5.0-6.0	5.5	1.3
17-	5755	14	5.7	5.2-6.2	5.5	1.4
18-	5607	13	5.9	5.3-6.4	5.4	1.4
19-	5121	15	6.1	5.5-6.6	5.1	1.5
20-	4292	17	6.3	5.8-6.9	4.4	1.6
21-	3555	14	6.6	6.0-7.2	3.8	1.7
22-	2931	9	6.8	6.2-7.4	3.3	1.7
23-	2334	13	7.2	6.6-7.8	2.7	1.8
24-	1776	7	7.4	6.8-8.0	2.2	1.9†
2000-2009						
0.5-	4637	5	0.1	0.0-0.1	3.1	0
1-	9168	18	0.2	0.1-0.4	6.3	0.1
2-	9013	27	0.5	0.4-0.7	6.2	0.2
3-	8843	25	0.8	0.6-1.0	6.1	0.2
4-	8699	31	1.2	0.9-1.4	6.1	0.3
5-	8536	23	1.4	1.2-1.6	6.1	0.4
6-	8362	36	1.8	1.5-2.1	6.0	0.4
7-	8184	29	2.1	1.8-2.4	5.9	0.5
8-	8013	20	2.3	2.0-2.6	5.9	0.6
9-	7394	18	2.6	2.2-2.9	5.5	0.7
10-	6391	18	2.8	2.4-3.1	4.8	0.7
11-	5436	12	3.0	2.6-3.3	4.2	0.8
12-	4501	10	3.1	2.8-3.5	3.5	0.8
13-	3639	9	3.3	2.9-3.7	2.9	0.9
14-	2892	6	3.5	3.1-3.9	2.3	1.0†
2010-2018						
0.5-	4913	6	0.1	0.0-0.1	2.9	0
1-	8753	11	0.2	0.1-0.3	5.2	0.1
2-	7391	9	0.3	0.2-0.4	4.5	0.1
3-	6028	13	0.5	0.3-0.7	3.7	0.2
4-	4711	13	0.8	0.6-1.0	3.0	0.3†

CI: confidence interval
*Risks given for the end of each period, i.e. risks in the line '9-' refer to 10 years after diagnosis, etc
†Risks at 25, 15, and 5 years after diagnosis of DCIS respectively

Supplementary Table S13. Characteristics of 9679 women with non-screen-detected unilateral ductal carcinoma in situ (DCIS) at ages 50-64 years, and 31,141 women with screen-detected unilateral DCIS at ages 50-64 DCIS, diagnosed in England during 1990-2018 and their status on December 2018.

	**Non-screen-detected DCIS**	**Screen-detected DCIS**	**P-val***	**Total**
Year of DCIS diagnosis				
1990-99	4101 (42.4)	3519 (11.3)	<0.001	7620 (18.7)
2000-09	2714 (28.0)	13,458 (43.2)		16,172 (39.6)
2010-18	2864 (29.6)	14,164 (45.5)		17,028 (41.7)
Age at DCIS diagnosis (years)				
50-54	3909 (40.4)	11,904 (38.2)	<0.001	15,813 (38.7)
55-59	2999 (31.0)	9458 (30.4)		12,457 (30.5)
60-64	2771 (28.6)	9779 (31.4)		12,550 (30.7)
Laterality of DCIS				
Left	4857 (52.4)	15,880 (51.7)	0.23	20,737 (51.9)
Right	4404 (47.6)	14,820 (48.3)		19,224 (48.1)
Bilateral†	71 (-)	114 (-)		185 (-)
Unknown†	347 (-)	327 (-)		674 (-)
Index of multiple deprivation				
1 – Most deprived	842 (14.0)	3696 (13.1)	0.43	4538 (13.2)
2	1015 (16.8)	4806 (17.0)		5821 (16.9)
3	1221 (20.3)	5864 (20.7)		7085 (20.6)
4	1423 (23.6)	6724 (23.7)		8147 (23.7)
5 - Least deprived	1525 (25.3)	7228 (25.5)		8753 (25.5)
Pre-1999‡	3639 (-)	2816 (-)		6455 (-)
Unknown	14 (-)	7 (-)		21 (-)
Length of follow-up since DCIS diagnosis (years)				
0-4	1904 (19.7)	8308 (26.7)		10,212 (25.0)
5-9	1763 (18.2)	8232 (26.4)		9995 (24.5)
10-19	3243 (33.5)	12,403 (39.8)		15,646 (38.3)
20-29	2769 (28.6)	2198 (7.1)		4967 (12.2)
Invasive breast cancer diagnosed by 31 Dec 2018				
Contralateral	458 (4.8)	1134 (3.7)		1592 (3.9)
Ipsilateral	718 (7.6)	1300 (4.2)		2018 (5.0)
Unknown	210 (-)	190 (-)		400 (-)
Vital status on 31 Dec 2018				
Alive	7726 (79.8)	28,209 (90.6)		35,935 (88.0)
Emigrated	188 (1.9)	383 (1.2)		571 (1.4)
Dead	1765 (18.2)	2549 (8.2)		4314 (10.6)
Death cause (% of total death)				
Breast cancer	335 (19.0)	403 (15.8)		738 (17.1)
Other causes	1396 (79.1)	2117 (83.1)		3513 (81.4)
Unknown cause	34 (1.9)	29 (1.1)		63 (1.5)
Total	9679 (100.0)	31,141 (100.0)		40,820 (100.0)

***P*-val for heterogeneity test

† Bilateral and unknown excluded from heterogeneity test

‡ Values of the index of multiple deprivation were not available for women diagnosed prior to 1999

Supplementary Table S14. Invasive breast cancer: Ratios of invasive breast cancer incidence rate in women with non-screen-detected DCIS compared with women with screen-detected DCIS. 9679 women aged 50-64 years with unilateral non-screen-detected ductal carcinoma in situ (DCIS) and 31,141 women with screen-detected DCIS in England

Time since diagnosis (years)	1990-1999 Non-screen-detected	Screen-detected	Ratio (95% CI)	2000-2009 Non-screen-detected	Screen-detected	Ratio (95% CI)	2010-2018 Non-screen-detected	Screen-detected	Ratio (95% CI)
0.5-	9/2046.26	11/1755.68	0.70 (0.29-1.64)	15/1351.21	26/6718.87	2.87 (1.52-5.42)	21/1371.40	21/6859.82	5.00 (2.73-9.16)
1-	37/4056.90	33/3479.48	0.96 (0.60-1.54)	26/2672.78	93/13351.46	1.40 (0.90-2.16)	31/2425.15	82/12308.06	1.92 (1.27-2.90)
2-	56/3993.36	33/3433.65	1.46 (0.95-2.24)	31/2632.78	121/13195.74	1.28 (0.87-1.91)	40/2038.01	96/10453.45	2.14 (1.48-3.09)
3-	40/3924.67	33/3389.51	1.05 (0.66-1.66)	38/2587.16	139/13004.69	1.37 (0.96-1.97)	32/1640.26	89/8631.56	1.89 (1.26-2.83)
4-	53/3857.71	38/3338.85	1.21 (0.80-1.83)	27/2540.35	114/12838.45	1.20 (0.79-1.82)	24/1263.94	76/6859.35	1.71 (1.08-2.71)
0.5-4	195/17878.90	148/15397.16	1.13 (0.92-1.40)	137/11784.27	493/59109.21	1.39 (1.15-1.68)	148/8738.76	364/45112.23	2.10 (1.73-2.54)
5-9	216/18235.73	180/15858.29	1.04 (0.86-1.27)	153/11902.42	615/60651.41	1.27 (1.06-1.51)			
10-14	182/16386.97	134/14428.10	1.20 (0.96-1.49)	83/7190.07	334/34364.41	1.19 (0.93-1.51)			
15-24	217/21885.30	170/17567.21	1.02 (0.84-1.25)						
Total	810/74385.90	632/63250.75	1.08 (0.98-1.21)	373/30876.76	1442/154125.00	1.29 (1.15-1.45)	148/8738.76	364/45112.23	2.10 (1.73-2.54)

CI: confidence interval

*Overall ratio (non-screen-detected: screen-detected) of incidence rates adjusted for calendar period and time since diagnosis, 1.26 (1.17-1.35) p<0.001

Trend test for calendar period adjusted for time since diagnosis: p<0.001

Trend test for time since diagnosis adjusted for calendar period: p<0.001

P for interaction between time since DCIS diagnosis and age at diagnosis: p=0.53

Supplementary Table S15. Breast cancer death: Ratios of breast cancer death rate in women with non-screen-detected DCIS compared with women with screen-detected DCIS. 9679 women aged 50-64 years with non-screen-detected unilateral ductal carcinoma in situ (DCIS) and 31,141 women with screen-detected DCIS in England

Time since diagnosis (years)	1990-1999 Non-screen-detected	Screen-detected	Ratio (95% CI)	2000-2009 Non-screen-detected	Screen-detected	Ratio (95% CI)	2010-2018 Non-screen-detected	Screen-detected	Ratio (95% CI)
0.5-	0/2048.68	3/1758.10	-	2/1354.47	3/6723.43	3.31 (0.55-19.81)	0/1378.77	1/6865.17	-
1-	8/4082.78	1/3506.39	6.87 (0.86-54.93)	1/2700.88	7/13417.70	0.71 (0.09-5.77)	1/2461.90	4/12368.87	1.26 (0.14-11.24)
2-	10/4055.94	4/3490.40	2.15 (0.67-6.86)	4/2687.41	9/13367.19	2.21 (0.68-7.18)	2/2097.07	2/10586.72	5.05 (0.71-35.84)
3-	11/4022.78	9/3471.46	1.05 (0.44-2.55)	5/2670.45	8/13312.13	3.12 (1.02-9.52)	1/1712.94	3/8821.42	1.72 (0.18-16.50)
4-	6/3998.23	7/3447.79	0.74 (0.25-2.20)	6/2649.34	11/13250.08	2.73 (1.01-7.38)	5/1345.16	3/7063.08	8.75 (2.09-36.62)
0.5-4	35/18208.42	24/15674.13	1.26 (0.75-2.11)	18/12062.55	38/60070.54	2.36 (1.35-4.13)	9/8995.84	13/45705.26	3.52 (1.50-8.23)
5-9	72/19346.54	45/16793.07	1.39 (0.96-2.02)	37/12761.55	81/64185.99	2.30 (1.56-3.39)			
10-14	51/18132.52	47/15869.77	0.95 (0.64-1.41)	17/8043.88	62/37702.16	1.29 (0.75-2.20)			
15-24	82/25345.42	62/20115.35	1.05 (0.75-1.46)						
Total	240/81032.90	178/68452.33	1.14 (0.94-1.38)	72/32869.98	181/161958.70	1.96 (1,49-2.57)	9/8995.84	13/45705.26	3.52 (1.50-8.23)

CI: confidence interval

* Overall ratio (non-screen-detected: screen-detected) of mortality rates adjusted for calendar period and time since, 1.37 (1.17-1.60) p=0.0001

Trend test for calendar period adjusted for time since diagnosis: p<0.001

Trend test for time since diagnosis adjusted for calendar period: p<0.001

P for interaction between time since DCIS diagnosis and age at diagnosis: p=0.40

Supplementary Table S16. Values used in figure 4 for comparison of cumulative risks of invasive breast cancer between non-screen-detected and screen-detected DCIS in women aged 50-64 yrs, by year of diagnosis and time since diagnosis. Cumulative risks take into account competing risks from other causes of death.

	Non-screen-detected			Screen-detected		
**Time since DCIS diagnosis (years)**	**Number of woman-years**	**Number of IBC observed**	**Cumulative observed risk (95% CI)***	**Number of woman-years**	**Number of IBC observed**	**Cumulative observed risk (95% CI)***
1990-1999						
0.5-	2046	9	0.2 (0.1- 0.4)	1756	11	0.3 (0.1- 0.5)
1-	4057	37	1.1 (0.8- 1.4)	3479	33	1.3 (0.9- 1.6)
2-	3993	56	2.5 (2.0- 3.0)	3434	33	2.2 (1.7- 2.7)
3-	3925	40	3.5 (2.9- 4.0)	3390	33	3.1 (2.6- 3.7)
4-	3858	53	4.8 (4.1- 5.4)	3339	38	4.2 (3.5- 4.9)
5-	3785	50	6.0 (5.3- 6.7)	3288	33	5.1 (4.4- 5.9)
6-	3720	38	6.9 (6.1- 7.7)	3227	36	6.2 (5.4- 7.0)
7-	3648	35	7.8 (7.0- 8.6)	3174	34	7.1 (6.3- 8.0)
8-	3582	51	9.0 (8.2- 9.9)	3119	43	8.4 (7.4- 9.3)
9-	3501	42	10.1 (9.1-11.0)	3051	34	9.3 (8.4-10.3)
10-	3424	48	11.2 (10.3-12.2)	2996	33	10.3 (9.3-11.3)
11-	3346	32	12.0 (11.0-13.0)	2940	23	10.9 (9.9-11.9)
12-	3281	31	12.8 (11.8-13.8)	2885	29	11.7 (10.7-12.8)
13-	3208	37	13.7 (12.6-14.8)	2832	13	12.1 (11.0-13.2)
14-	3126	34	14.5 (13.5-15.6)	2776	36	13.1 (12.0-14.2)
15-	3053	27	15.2 (14.1-16.3)	2708	23	13.8 (12.6-14.9)
16-	2971	36	16.1 (15.0-17.2)	2648	22	14.4 (13.2-15.5)
17-	2885	30	16.8 (15.7-18.0)	2583	27	15.1 (14.0-16.3)
18-	2803	30	17.6 (16.4-18.7)	2509	26	15.9 (14.7-17.1)
19-	2572	20	18.1 (16.9-19.3)	2184	21	16.5 (15.3-17.7)
20-	2171	15	18.5 (17.3-19.7)	1674	15	17.1 (15.9-18.4)
21-	1802	19	19.2 (18.0-20.4)	1252	15	17.9 (16.6-19.2)
22-	1498	16	19.8 (18.6-21.1)	900	13	18.7 (17.4-20.1)
23-	1208	12	20.4 (19.1-21.7)	643	6	19.3 (17.8-20.7)
24-	922	12	21.1 (19.8-22.4)	465	2	19.5 (18.0-20.9) †
2000-2009						
0.5-	1351	15	0.6 (0.3- 0.8)	6719	26	0.2 (0.1- 0.3)
1-	2673	26	1.5 (1.1- 2.0)	13351	93	0.9 (0.7- 1.0)
2-	2633	31	2.7 (2.1- 3.3)	13196	121	1.8 (1.6- 2.0)
3-	2587	38	4.1 (3.3- 4.8)	13005	139	2.8 (2.5- 3.1)
4-	2540	27	5.1 (4.2- 5.9)	12838	114	3.7 (3.3- 4.0)
5-	2497	37	6.4 (5.5- 7.4)	12641	150	4.8 (4.4- 5.1)
6-	2455	28	7.5 (6.5- 8.5)	12451	92	5.5 (5.1- 5.8)
7-	2403	36	8.8 (7.7- 9.9)	12278	95	6.2 (5.8- 6.6)
8-	2358	25	9.7 (8.6-10.8)	12082	190	7.6 (7.1- 8.0)
9-	2189	27	10.8 (9.6-11.9)	11200	88	8.3 (7.8- 8.7)
10-	1931	19	11.6 (10.4-12.8)	9722	83	9.0 (8.5- 9.5)
11-	1674	24	12.8 (11.5-14.1)	8200	104	10.1 (9.5-10.6)
12-	1424	18	13.8 (12.4-15.1)	6784	48	10.6 (10.1-11.2)
13-	1187	12	14.6 (13.2-16.0)	5455	46	11.3 (10.7-11.9)
14-	974	10	15.4 (13.9-16.8)	4205	53	12.3 (11.7-12.9) †
2010-2018						
0.5-	1371	21	0.8 (0.4-1.1)	6860	21	0.2 (0.1-0.2)
1-	2425	31	2.0 (1.5-2.6)	12308	82	0.8 (0.7-1.0)
2-	2038	40	3.9 (3.1-4.7)	10453	96	1.7 (1.5-2.0)
3-	1640	32	5.7 (4.8-6.7)	8632	89	2.7 (2.4-3.0)
4-	1264	24	7.5 (6.3-8.7)	6859	76	3.8 (3.4-4.2) †

CI: confidence interval

*Risks given for the end of each period, i.e. risks in the line '9-' refer to 10 years after diagnosis, etc

†Risks at 25, 15, and 5 years after diagnosis of DCIS respectively

Supplementary Table S17. Values used in figure 4 for comparison of cumulative risks of breast cancer death between non-screen-detected and screen-detected DCIS in women aged 50-64 yrs, by year of diagnosis and time since diagnosis. Cumulative risks take into account competing risks from other causes of death.

	Non-screen-detected			Screen-detected		
**Time since DCIS diagnosis (years)**	**Number of woman-years**	**Number of IBC observed**	**Cumulative observed risk (95% CI)***	**Number of woman-years**	**Number of IBC observed**	**Cumulative observed risk (95% CI)***
1990-1999						
0.5-	2049	0	0.0 (0.0-0.0)	1758	3	0.1 (0.0-0.2)
1-	4083	8	0.2 (0.1-0.3)	3506	1	0.1 (0.0-0.2)
2-	4056	10	0.4 (0.2-0.6)	3490	4	0.2 (0.1-0.4)
3-	4023	11	0.7 (0.5-1.0)	3471	9	0.5 (0.3-0.7)
4-	3998	6	0.9 (0.6-1.1)	3448	7	0.7 (0.4-1.0)
5-	3958	18	1.3 (0.9-1.6)	3424	10	1.0 (0.6-1.3)
6-	3914	15	1.7 (1.3-2.1)	3390	10	1.2 (0.9-1.6)
7-	3870	13	2.0 (1.6-2.4)	3359	9	1.5 (1.1-1.9)
8-	3826	15	2.4 (1.9-2.8)	3327	7	1.7 (1.3-2.1)
9-	3779	11	2.6 (2.1-3.1)	3292	9	2.0 (1.5-2.4)
10-	3733	7	2.8 (2.3-3.3)	3255	10	2.2 (1.8-2.7)
11-	3679	12	3.1 (2.6-3.6)	3215	9	2.5 (2.0-3.0)
12-	3631	14	3.4 (2.9-4.0)	3177	13	2.9 (2.3-3.4)
13-	3574	9	3.7 (3.1-4.2)	3133	2	2.9 (2.4-3.5)
14-	3516	9	3.9 (3.3-4.5)	3090	13	3.3 (2.7-3.9)
15-	3458	10	4.1 (3.5-4.7)	3043	5	3.4 (2.8-4.0)
16-	3387	11	4.4 (3.8-5.0)	2991	5	3.6 (3.0-4.2)
17-	3321	6	4.5 (3.9-5.2)	2932	10	3.9 (3.2-4.5)
18-	3254	11	4.8 (4.2-5.5)	2873	9	4.1 (3.5-4.8)
19-	2994	10	5.1 (4.4-5.8)	2513	11	4.4 (3.8-5.1)
20-	2538	8	5.3 (4.6-6.0)	1931	7	4.7 (4.0-5.4)
21-	2111	5	5.5 (4.8-6.2)	1453	4	4.9 (4.2-5.7)
22-	1766	6	5.7 (5.0-6.5)	1063	4	5.2 (4.4-6.0)
23-	1428	9	6.2 (5.4-6.9)	769	4	5.5 (4.7-6.4)
24-	1087	6	6.5 (5.7-7.3)	549	3	5.9 (5.0-6.8) †
2000-2009						
0.5-	1354	2	0.1 (0.0-0.2)	6723	3	0.0 (0.0-0.0)
1-	2701	1	0.1 (0.0-0.2)	13418	7	0.1 (0.0-0.1)
2-	2687	4	0.3 (0.1-0.4)	13367	9	0.1 (0.1-0.2)
3-	2670	5	0.4 (0.2-0.7)	13312	8	0.2 (0.1-0.3)
4-	2649	6	0.7 (0.4-1.0)	13250	11	0.3 (0.2-0.4)
5-	2628	6	0.9 (0.5-1.2)	13169	17	0.4 (0.3-0.5)
6-	2607	9	1.2 (0.8-1.6)	13075	15	0.5 (0.4-0.6)
7-	2580	8	1.5 (1.1-2.0)	12975	13	0.6 (0.5-0.7)
8-	2551	9	1.8 (1.3-2.4)	12889	17	0.7 (0.6-0.9)
9-	2395	5	2.0 (1.5-2.6)	12079	19	0.9 (0.7-1.0)
10-	2125	3	2.2 (1.6-2.7)	10535	15	1.0 (0.9-1.2)
11-	1864	4	2.4 (1.8-3.0)	8960	20	1.2 (1.0-1.4)
12-	1596	5	2.7 (2.0-3.3)	7478	11	1.4 (1.2-1.6)
13-	1342	2	2.8 (2.1-3.4)	6042	11	1.5 (1.3-1.8)
14-	1116	3	3.0 (2.3-3.7)	4688	5	1.6 (1.4-1.9) †
2010-2018						
0.5-	1379	0	0.0 (0.0-0.0)	6865	1	0.0 (0.0-0.0)
1-	2462	1	0.0 (0.0-0.1)	12369	4	0.0 (0.0-0.1)
2-	2097	2	0.1 (0.0-0.3)	10587	2	0.1 (0.0-0.1)
3-	1713	1	0.2 (0.0-0.4)	8821	3	0.1 (0.0-0.1)
4-	1345	5	0.6 (0.2-0.9)	7063	3	0.1 (0.1-0.2) †

CI: confidence interval
*Risks given for the end of each period, i.e. risks in the line '9-' refer to 10 years after diagnosis, etc
†Risks at 25, 15, and 5 years after diagnosis of DCIS respectively

Supplementary Table S18. Values used in figure 5 for cumulative rates and 95% confidence intervals in 22,753 women with non-screen-detected unilateral DCIS during 1990-2018

Ipsilateral IBC	Year 5	10	15	20	25
**BCS + RT**	3.2 (2.6- 3.8)	7.3 (6.3- 8.3)	11.6 (10.0-13.1)	15.0 (12.8-17.1)	19.8 (16.2-23.4)
**BCS no RT**	5.5 (5.0- 6.1)	10.4 (9.6-11.3)	14.6 (13.5-15.7)	17.9 (16.5-19.3)	20.6 (18.7-22.4)
**Mastectomy**	2.9 (2.5- 3.3)	5.2 (4.6- 5.7)	6.2 (5.5- 6.8)	7.2 (6.4- 8.0)	8.2 (7.0- 9.4)

$P_{heterogeneity}$ <0.001

Contralateral IBC	Year 5	10	15	20	25
**BCS + RT**	2.3 (1.8- 2.8)	4.2 (3.4- 4.9)	5.6 (4.6- 6.7)	7.7 (6.1- 9.2)	9.9 (7.4-12.3)
**BCS no RT**	1.8 (1.5- 2.1)	3.6 (3.1- 4.1)	6.5 (5.7- 7.3)	8.5 (7.5- 9.6)	10.6 (9.1-12.1)
**Mastectomy**	2.0 (1.7- 2.3)	4.2 (3.7- 4.7)	6.3 (5.6- 7.0)	8.5 (7.5- 9.5)	10.1 (8.6-11.5)

$P_{heterogeneity}$=0.94

Breast cancer death	Year 5	10	15	20	25
**BCS + RT**	0.9 (1.6- 2.7)	2.2 (3.7- 5.7)	4.7 (4.4- 7.0)	5.7 (6.2-10.9)	8.6 (5.9-15.5)
**BCS no RT**	0.7 (1.9- 2.6)	2.3 (3.0- 4.1)	3.5 (4.2- 5.7)	5.0 (6.4- 9.2)	7.8 (6.3-11.5)
**Mastectomy**	1.0 (2.0- 2.7)	2.3 (3.0- 4.0)	3.5 (4.4- 5.9)	5.1 (5.3- 7.8)	6.5 (4.9-10.9)

$P_{heterogeneity}$=0.57

BCS+RT: breast-conserving surgery, radiotherapy recorded
BCS no RT: breast-conserving surgery, radiotherapy not recorded

Supplementary Table S19. The distributions of clinical variables by calendar period (censored on 30 Dec 2018).

Category	1990-99	2000-09	2010-18	Total
Tumour size (mm)				
<=10	439 (5.5)	762 (8.2)	663 (6.5)	1864 (6.8)
11-20	562 (7.0)	829 (8.9)	709 (6.9)	2100 (7.6)
21-50	395 (4.9)	1129 (12.1)	1108 (10.8)	2632 (9.6)
51+	61 (0.8)	352 (3.8)	508 (5.0)	921 (3.3)
Unknown	6536 (81.8)	6242 (67.0)	7248 (70.8)	20,026 (72.7)
Grade				
High	378 (4.7)	1953 (21.0)	3155 (30.8)	5486 (19.9)
Intermediate	258 (3.2)	1051 (11.3)	1904 (18.6)	3213 (11.7)
Low	249 (3.1)	609 (6.5)	859 (8.4)	1717 (6.2)
Unknown	7108 (88.9)	5701 (61.2)	4318 (42.2)	17,127 (62.2)
Oestrogen receptor (ER) status and endocrine therapy				
ER+, endocrine	6 (0.1)	62 (0.7)	318 (3.1)	386 (1.4)
ER+, no endocrine	8 (0.1)	327 (3.5)	1840 (18.0)	2175 (7.9)
ER-	4 (0.1)	153 (1.6)	500 (4.9)	657 (2.4)
Unknown	7975 (99.8)	8772 (94.2)	7578 (74.0)	24,325 (88.3)
Total	7993 (100.0)	9314 (100.0)	10,236 (100.0)	27,543 (100.0)
